# Supplementary material for: Light-responsive and ultrapermeable two-dimensional metal-organic framework membrane for efficient ionic energy harvesting
Source: Nat Commun. 2024 Mar 8;15:2125. doi: 10.1038/s41467-024-46439-w (PMC10923900; doi:10.1038/s41467-024-46439-w)
Supplement: Supplementary file 1 — Supplementary Information [file 41467_2024_46439_MOESM1_ESM.pdf]

Supplementary Information

Light-Responsive and Ultrapermeable Two-Dimensional Metal-organic Framework Membrane for Efficient Ionic Energy Harvesting

Jin Wang[1*], Zeyuan Song[1], Miaolu He[1], Yongchao Qian[2], Di Wang[1], Zheng Cui[1], Yuan Feng[1], Shangzhen Li[1], Bo Huang[3], Xiangyu Kong[2*], Jinming Han[1], Lei Wang[1*]

1 Research Institute of Membrane Separation Technology of Shaanxi Province, Key Laboratory of Membrane Separation of Shaanxi Province, School of Environmental & Municipal Engineering, Xi'an University of Architecture and Technology, No. 13 Yan Ta Road, Xi'an 710000, China.

2. CAS Key Laboratory of Bio-inspired Materials and Interfacial Science, Technical Institute of Physics and Chemistry, Chinese Academy of Sciences, No. 29 Zhongguancun East Road, Beijing, 100190, China.

3 Institute of Chemical Engineering and Technology, Xi'an Jiaotong University, No. 28, West Xianning Road, Xi'an, 710049, China.

*E-mail: wangjin@xauat.edu.cn, kongxiangyu@mail.ipc.ac.cn, wl0178@126.com.

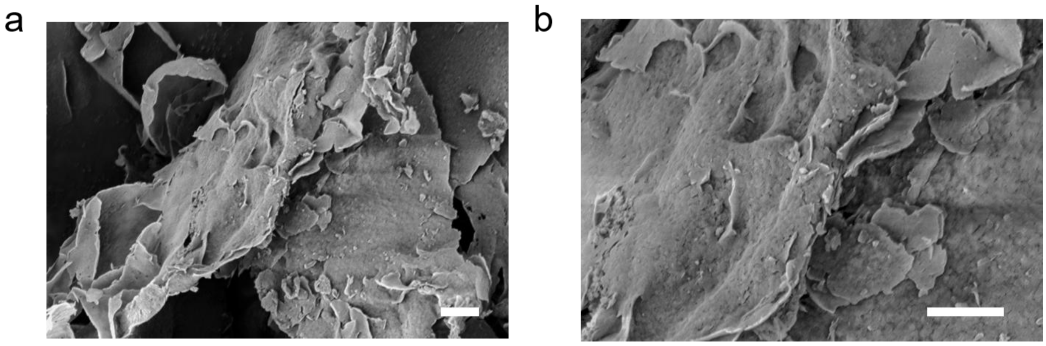

Supplementary Figure 1. SEM image of bulk Cu-TCPP crystal. Scale bar, 2 μm.

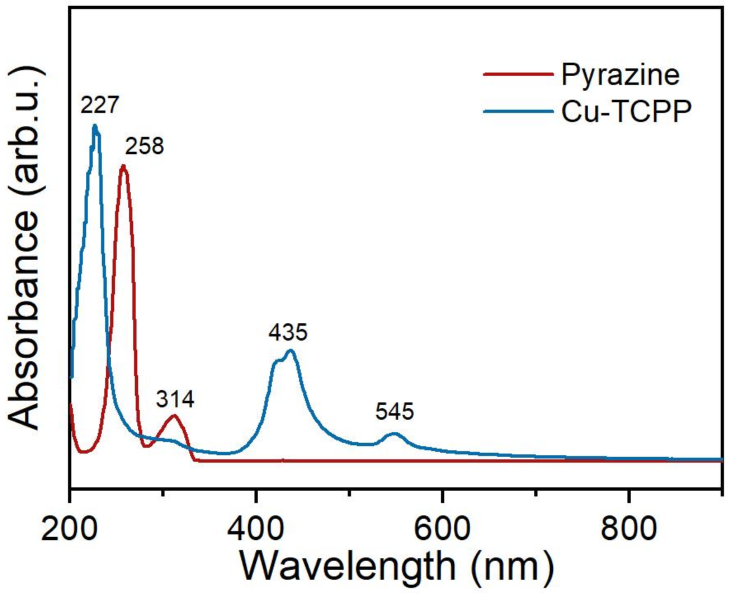

Supplementary Figure 2. UV-Vis spectra of pyrazine and Cu-TCPP dispersion.

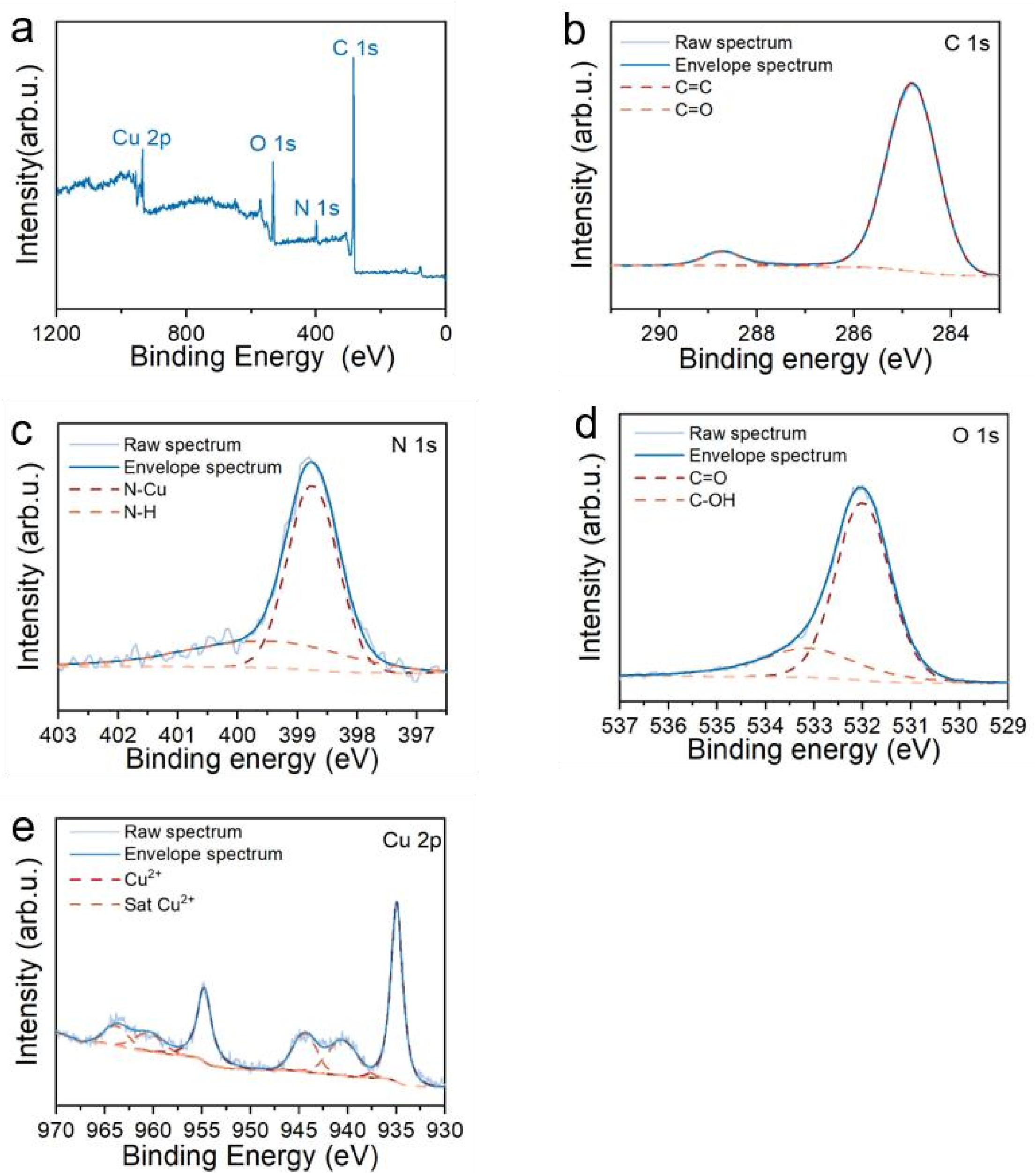

Supplementary Figure 3. XPS spectra of the Cu-TCPP membrane. (a~e) The XPS results and the C 1*s*, N 1*s*, O 1*s*, Cu 2*p* region of the Cu-TCPP membrane.

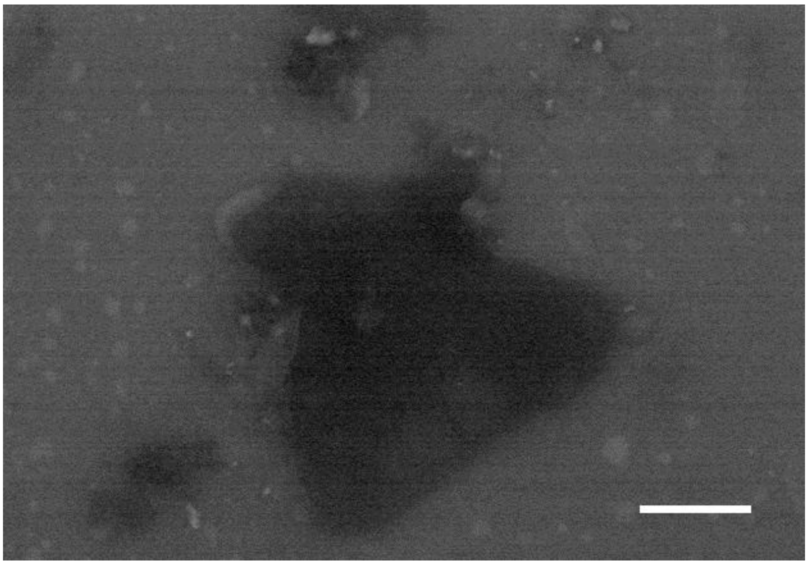

Supplementary Figure 4. SEM image of Cu-TCPP nanosheets. Scale bar, 1 μm.

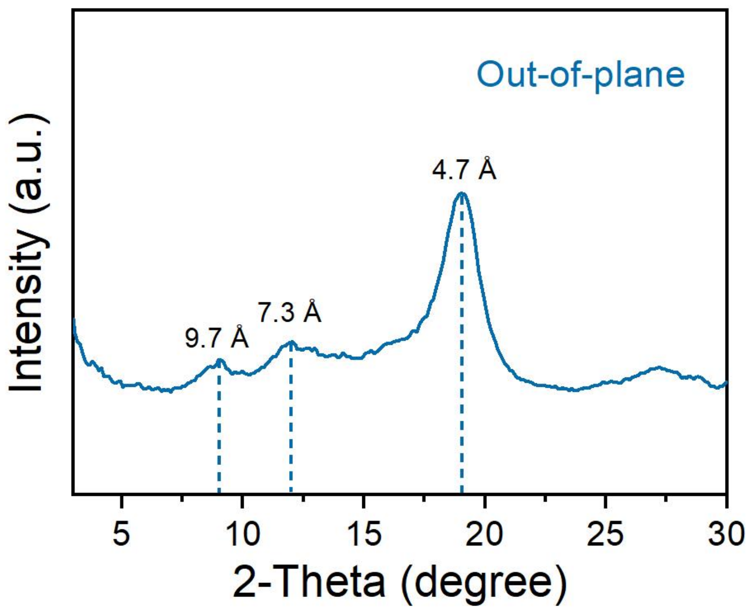

Supplementary Figure 5. Out-of-plane WAXS pattern of Cu-TCPP lamellar membrane.

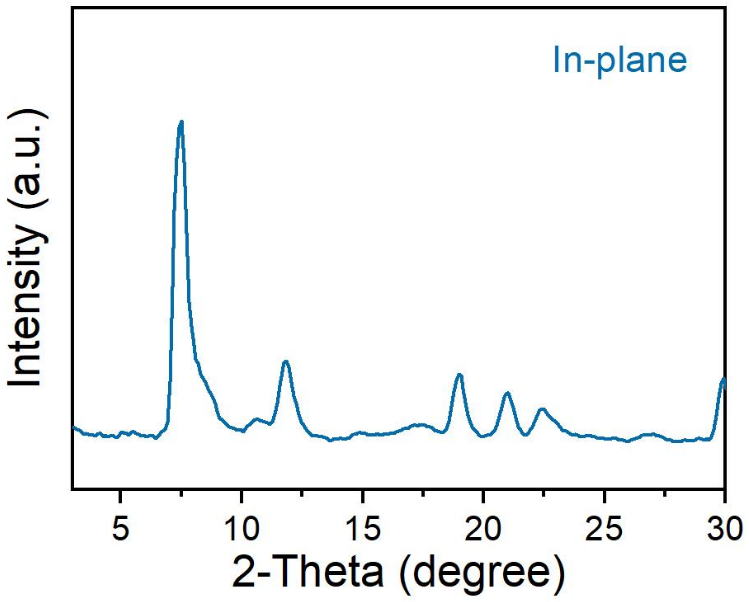

Supplementary Figure 6. In-plane WAXS pattern of Cu-TCPP lamellar membrane.

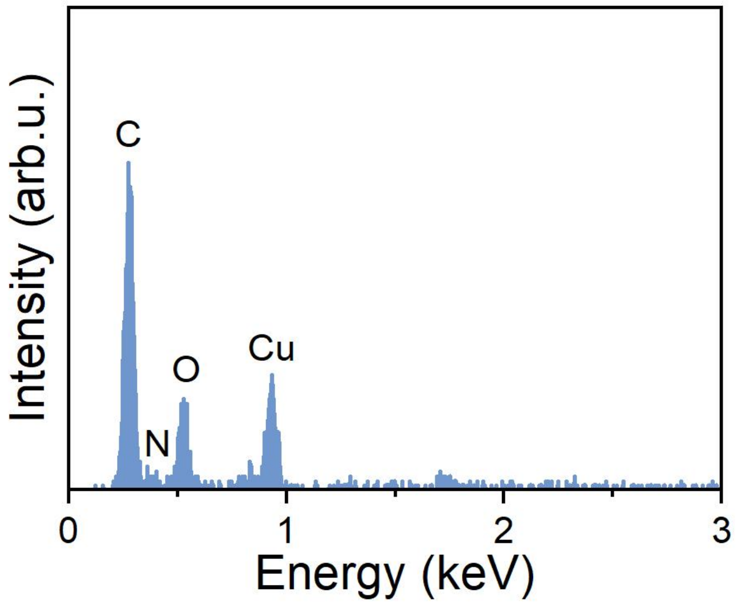

Supplementary Figure 7. EDS spectrum of the Cu-TCPP nanosheet.

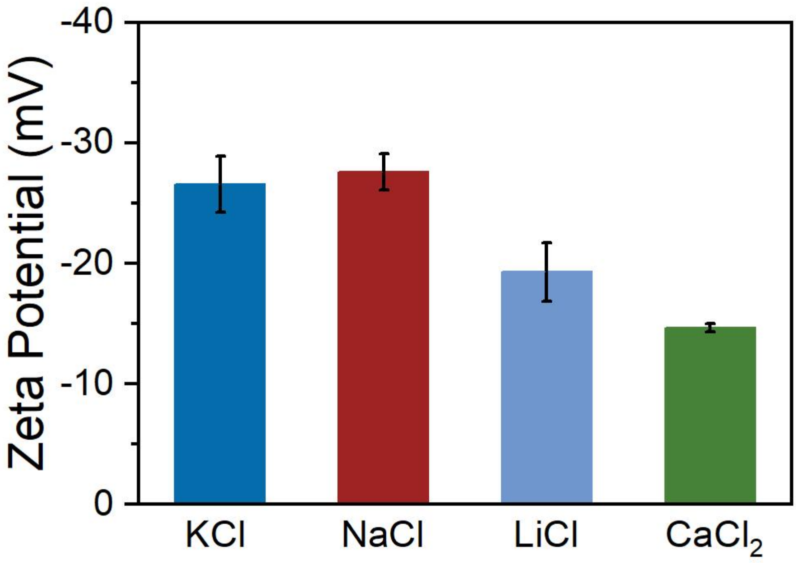

Supplementary Figure 8. Zeta potential values of Cu-TCPP nanosheet in different electrolyte solutions. The error bars represent the standard deviations.

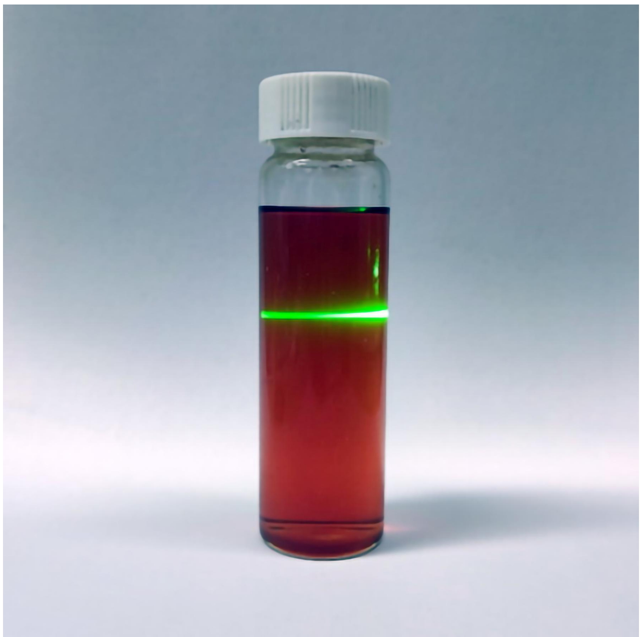

Supplementary Figure 9. The Tyndall phenomenon of Cu-TCPP nanosheet dispersion.

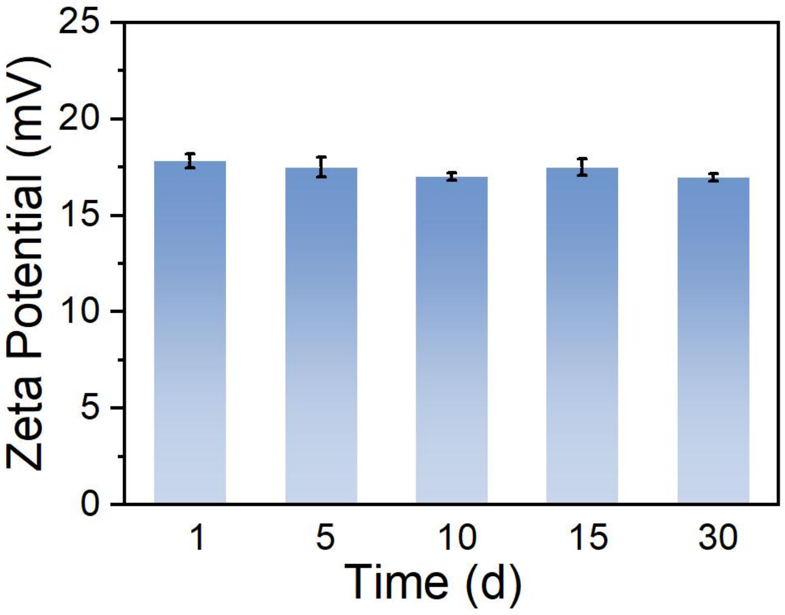

Supplementary Figure 10. Zeta potential changes during thirty days. The error bars represent the standard deviations.

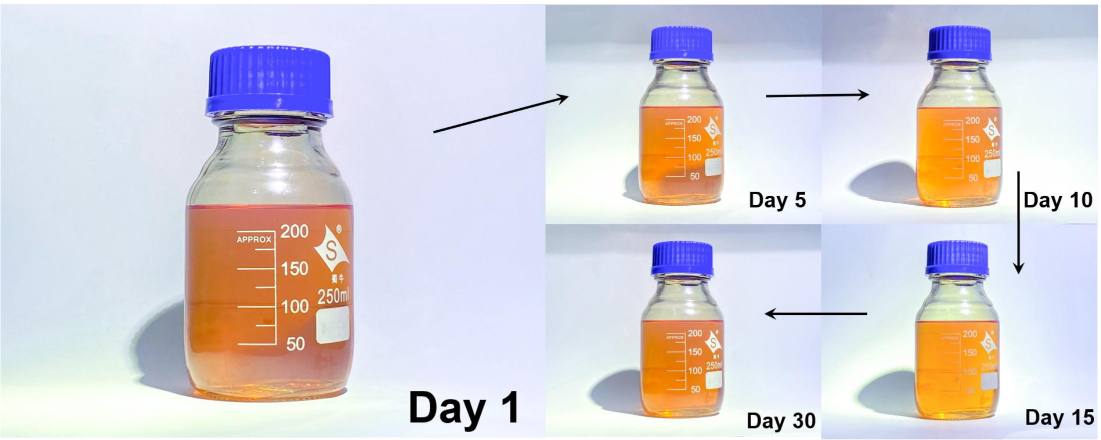

Supplementary Figure 11. Long-term stability of Cu-TCPP nanosheet dispersions.

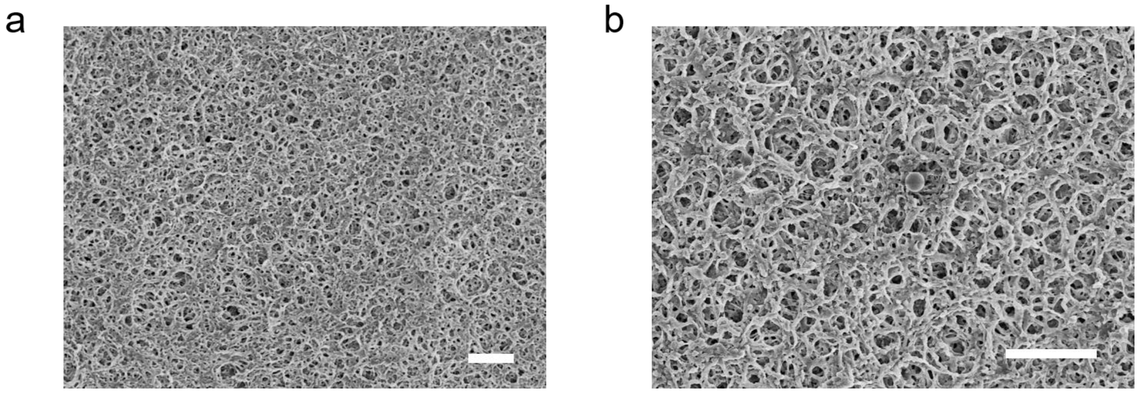

Supplementary Figure 12. Images of PVDF porous substrate at different magnifications. Scale bar, 10 μm.

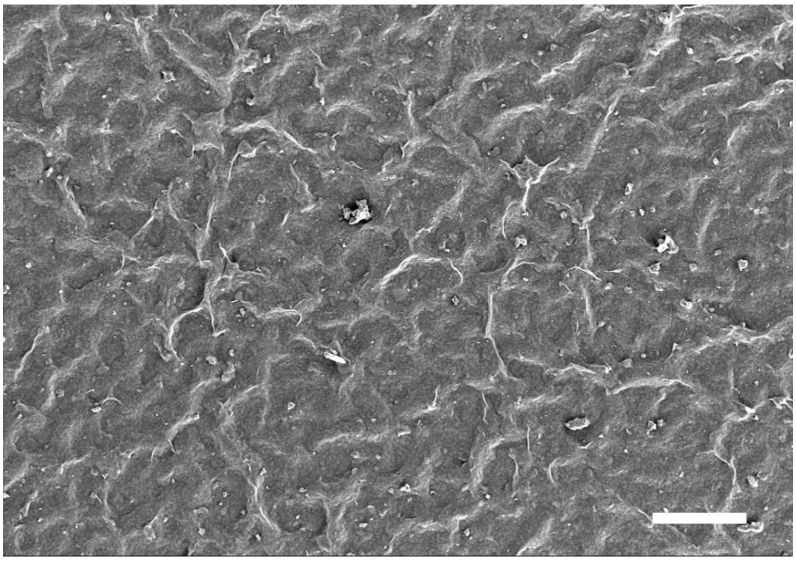

Supplementary Figure 13. Surface SEM image of the Cu-TCPP membranes. Scale bar, 10 μm.

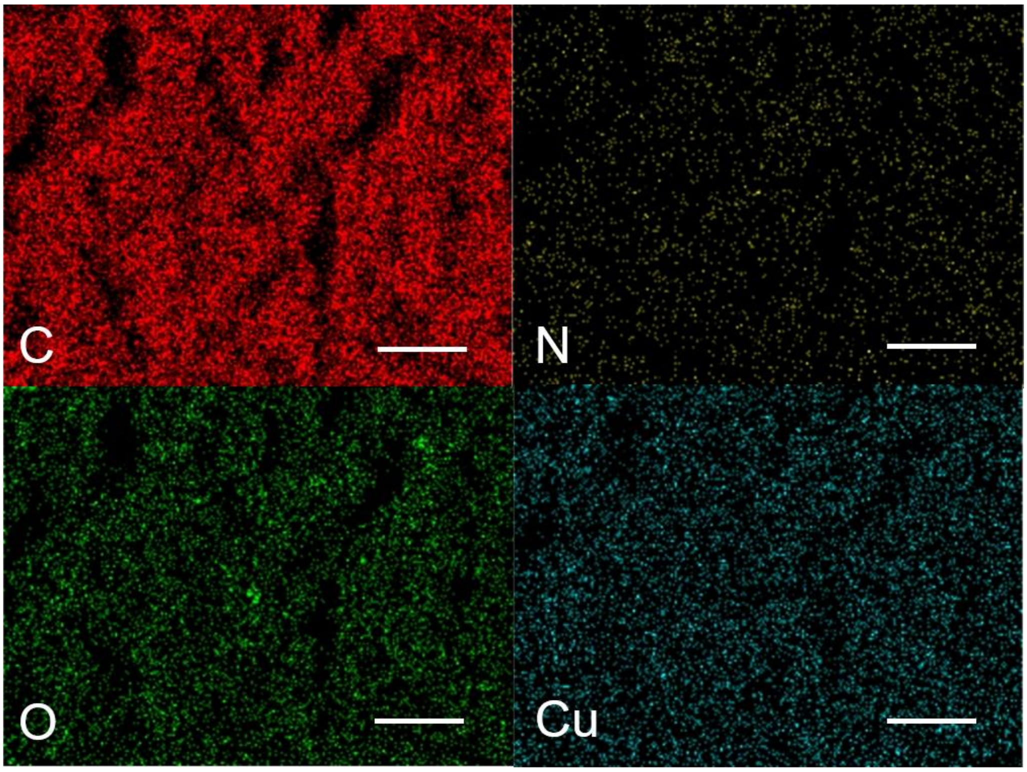

Supplementary Figure 14. Surface SEM mappings of Cu-TCPP membranes. Scale bar, 10 μm.

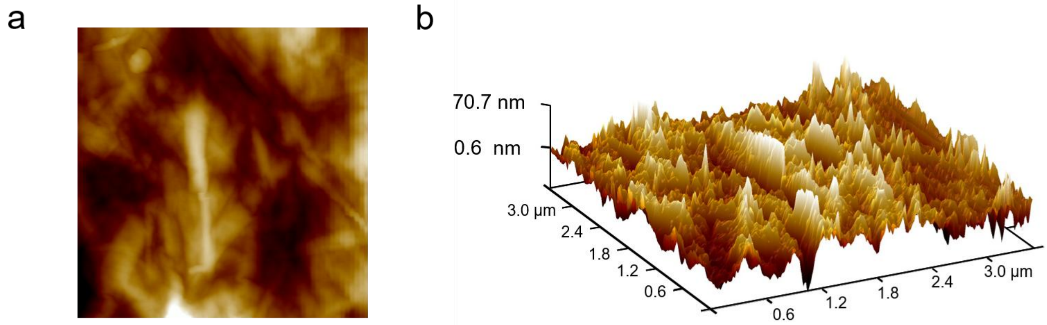

Supplementary Figure 15. AFM images of the Cu-TCPP membrane surface with a scan area of 3.5 µm × 3.5 µm. (a) 2D and (b) 3D AFM images.

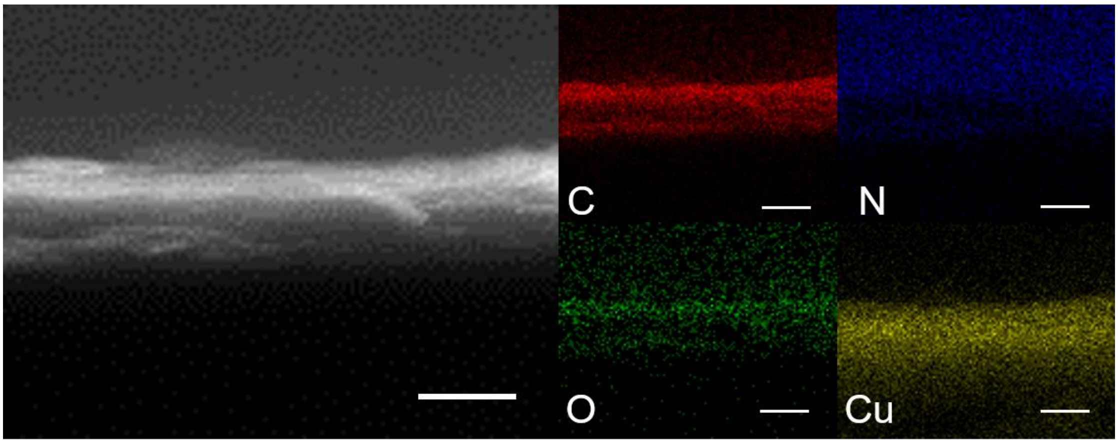

Supplementary Figure 16. SEM image of the Cu-TCPP membrane cross-section and elemental mapping. Scale bar, 15 μm.

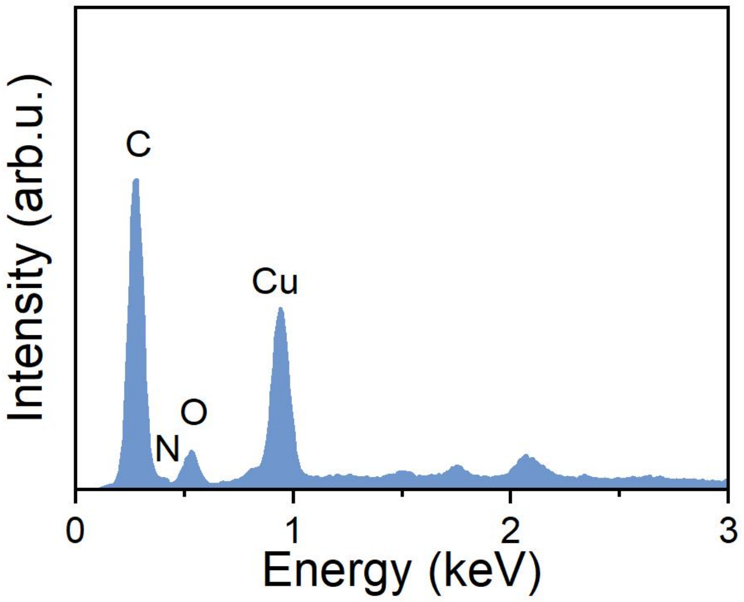

Supplementary Figure 17. EDS spectrum of the Cu-TCPP membrane cross-section.

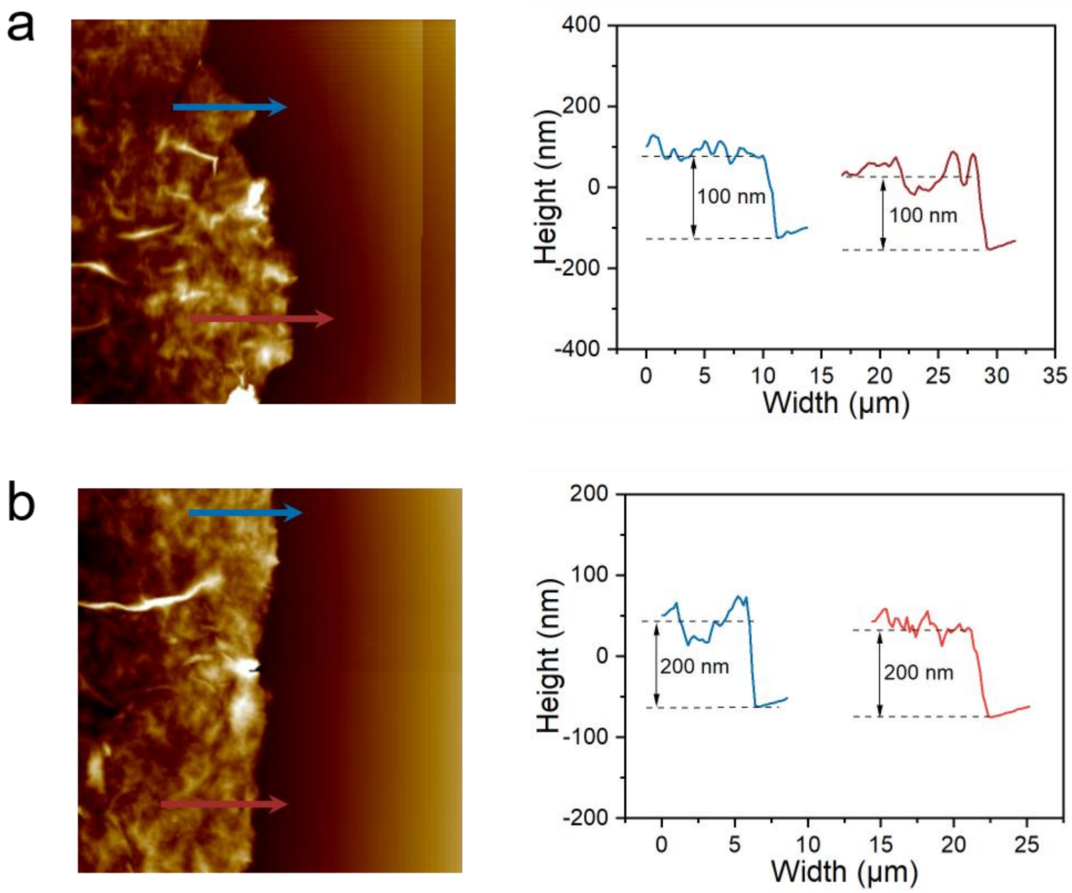

Supplementary Figure 18. AFM images of Cu-TCPP membranes with different thicknesses. The scanning direction is the direction of the arrow in the image.

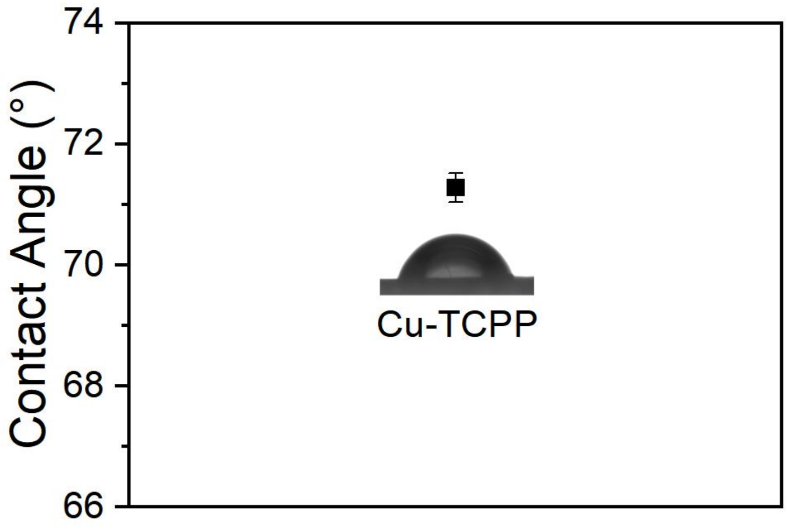

Supplementary Figure 19. The contact angle of water on the Cu-TCPP membrane surfaces. The error bars represent the standard deviations.

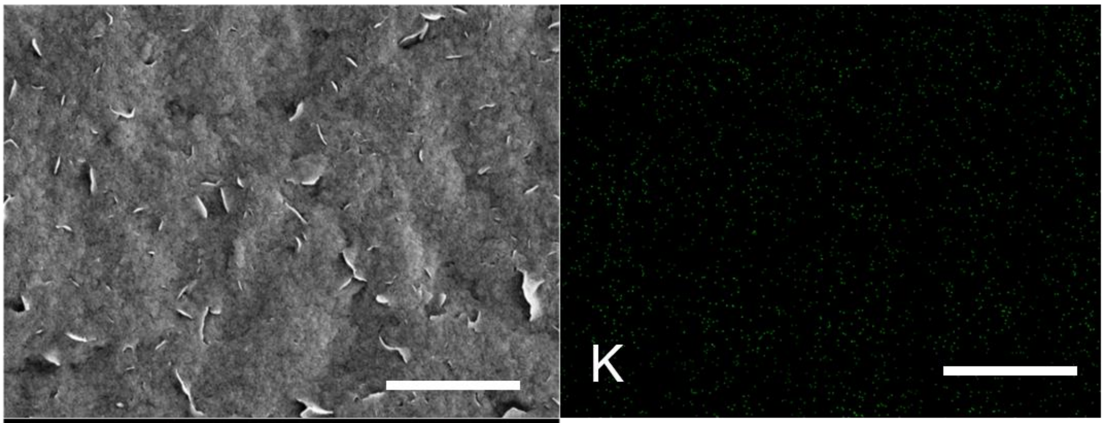

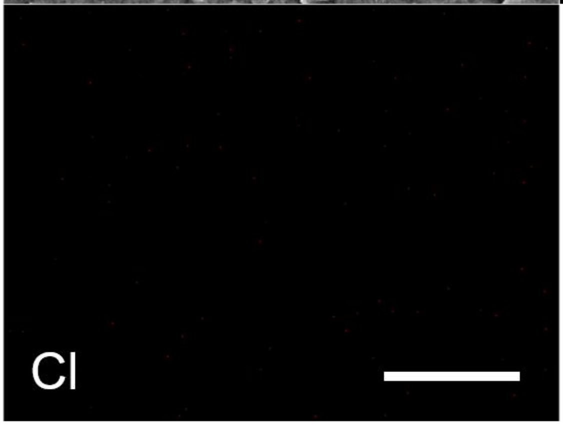

Elemental	Weight %	Atomic %
Cl	1.10	3.2
K	98.90	96.8

Supplementray Figure 20. EDS mappings images of K^+ and Cl^- and content information of membrane surface after immersing in 0.5 M KCl solution for 10 h. Scale bar, 10 μm.

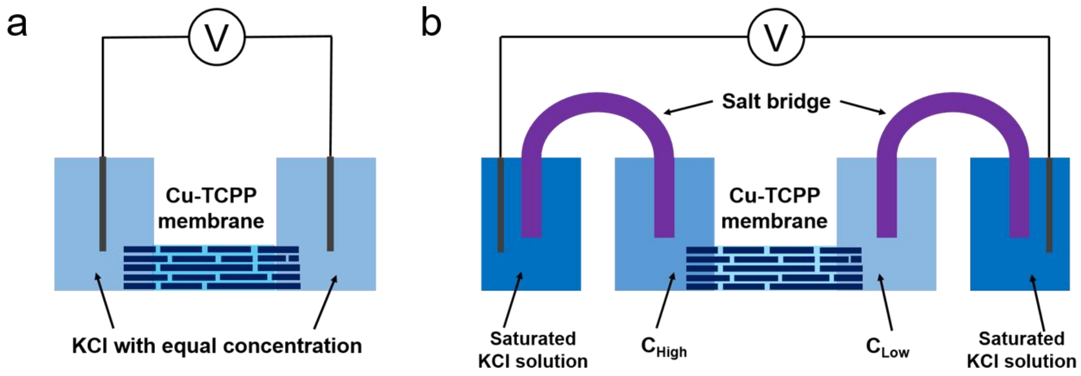

Supplementary Figure 21. Scheme of experimental setups. (a) Electrochemical cell for measuring I-V curves with identical electrolyte solutions placed on the two sides of the membrane. (b) The osmotic current and diffusion potential were derived from the I-V curves measured under the transmembrane concentration gradients. A pair of salt bridges eliminated the imbalanced electrode potential.

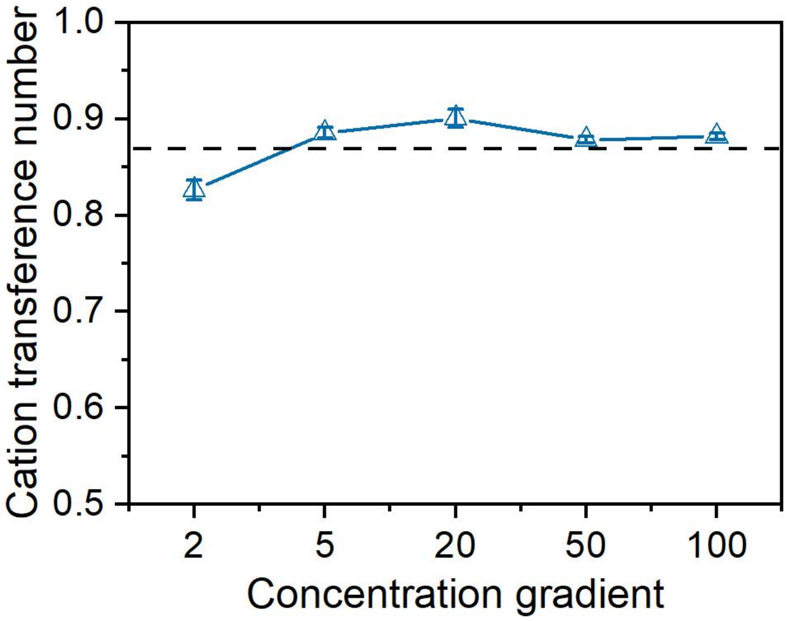

Supplementary Figure 22. Cation transfer number (t_+) of the Cu-TCPP membranes versus concentration gradient. The error bars represent the standard deviations.

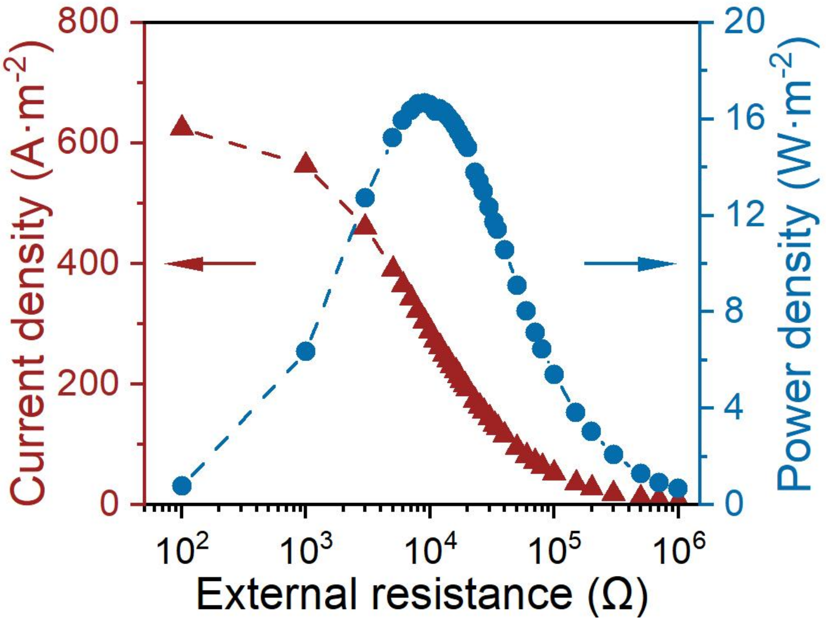

Supplementary Figure 23. The output power density and current density of Cu-TCPP membrane as functions of load resistance artificial seawater/river water system (NaCl solution 0.5 M/0.01 M**).**

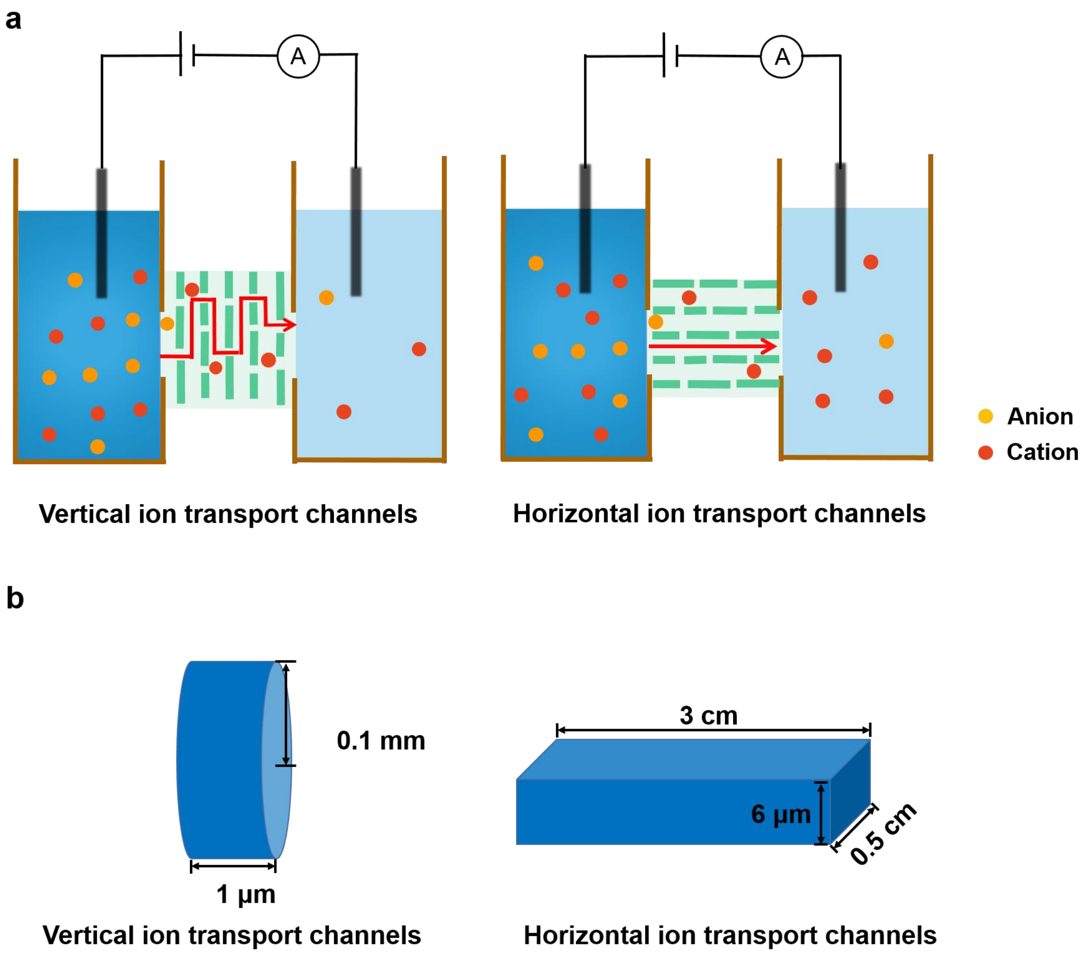

Supplementary Figure 24. Schematic illustration two different transport models (a) and the membrane size for ionic current comparison (b).

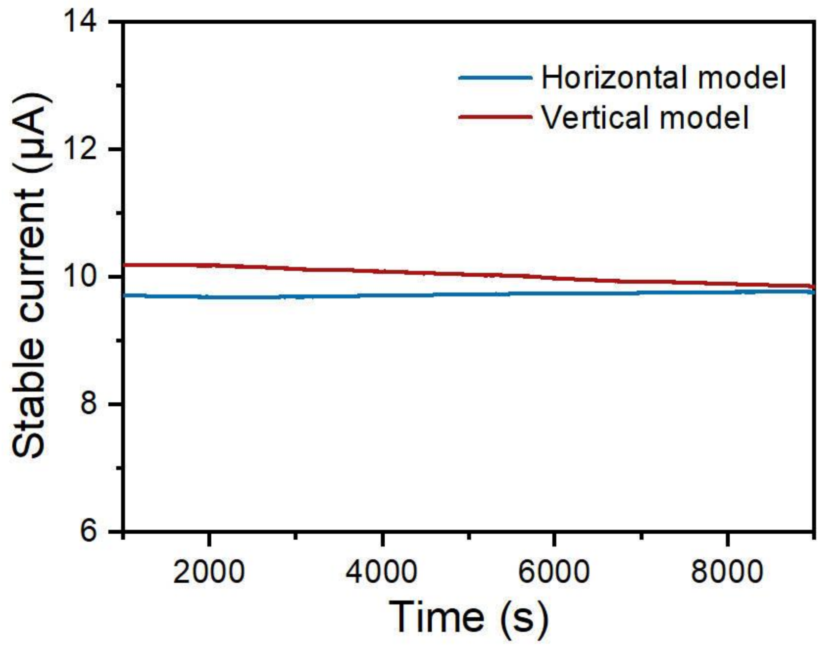

Supplementary Figure 25. Stabilized ionic currents in the horizontal and vertical transport model.

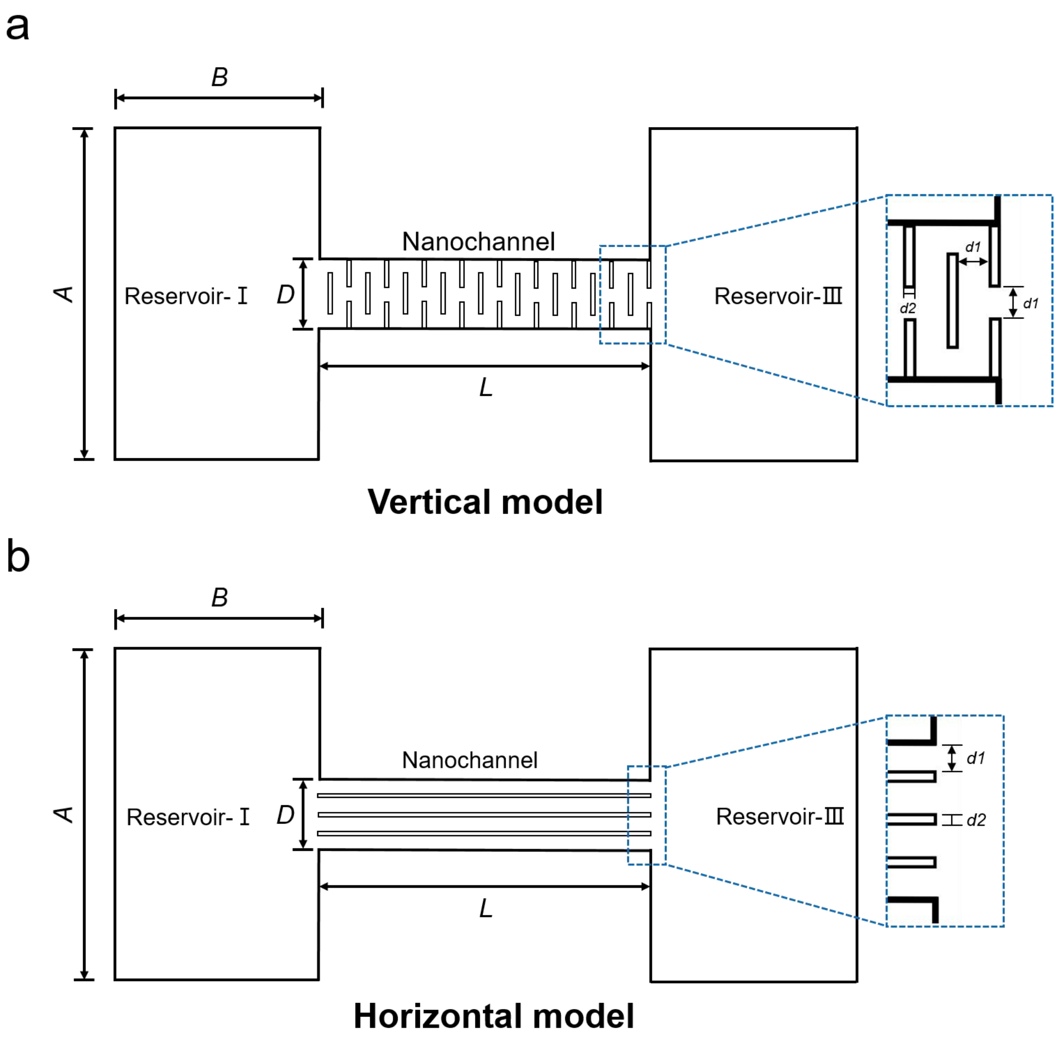

Supplementary Figure 26. Schematic illustration of vertical transportation model (a), horizontal transportation model (b).

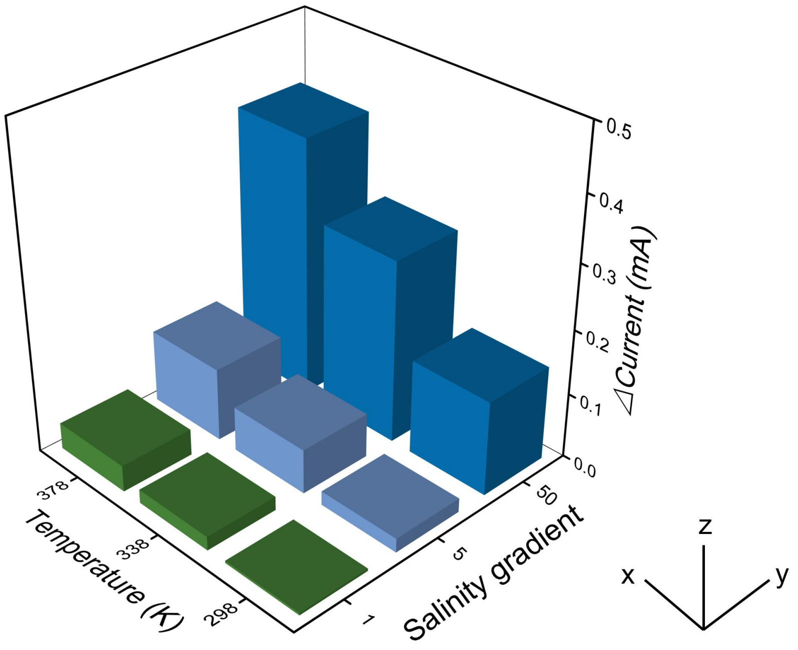

Supplementary Figure 27. The current difference at the orifice of the horizontal and vertical transport model under different salinity gradients/temperature gradients. X-axis represented the temperature of reservoir-III; y-axis represented the concentration of reservoir-I; and z-axis represented the current difference.

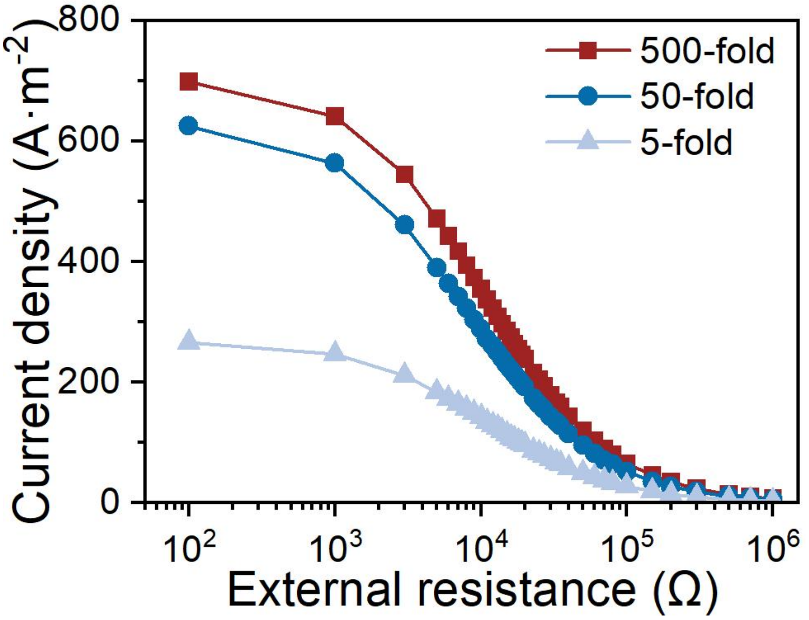

Supplementary Figure 28. The current density of Cu-TCPP membrane as functions of load resistance in different electrolyte solutions. The NaCl solution with different concentrations of 0.05~5 M was used in Reservoir-I, and 0.01M in Reservoir-III, respectively.

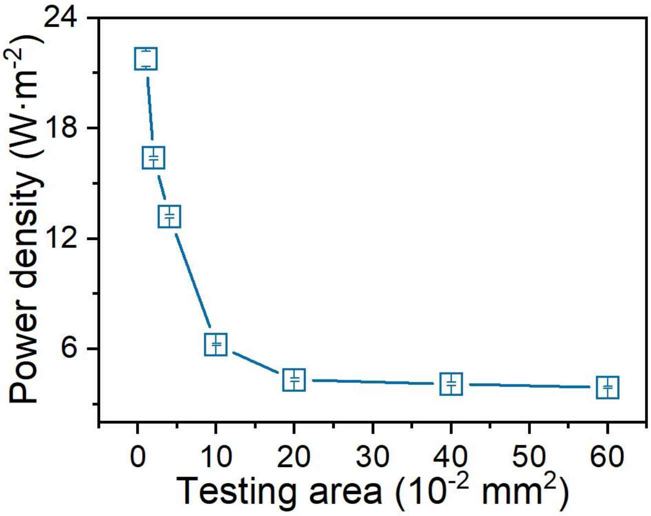

Supplementary Figure 29. Power density with different test area. Detailed membrane size parameters were shown in Supplementary Table 4. Error bars indicated the standard deviations from three different samples.

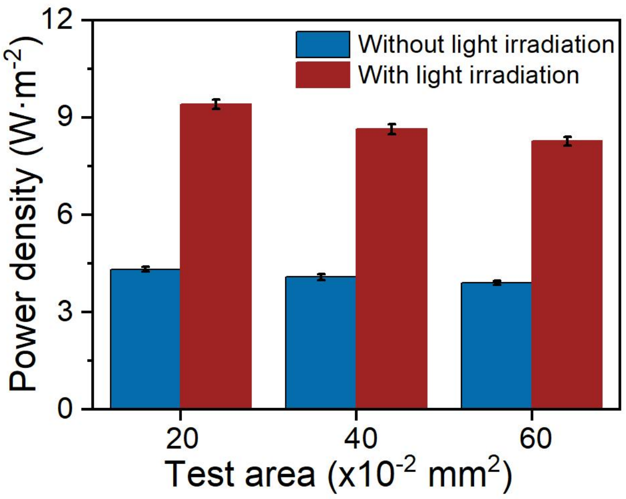

Supplementary Figure 30. Output power of different test areas under illuminated and non-illuminated conditions.

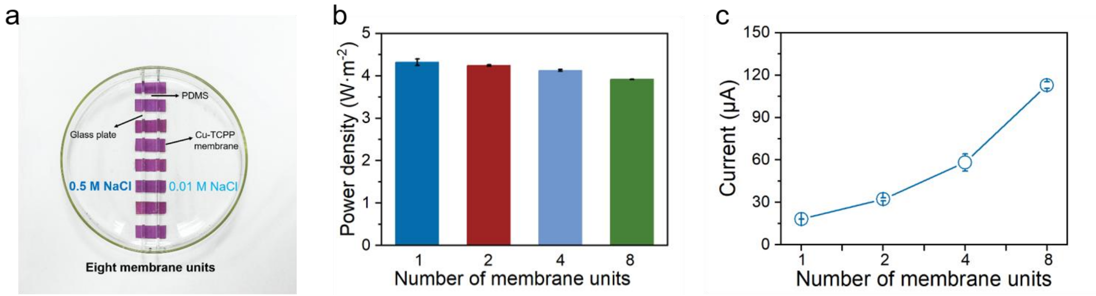

Supplementary Figure 31. Relationship between current, power density and number of Cu-TCPP-RED membrane units. Error bars indicated the standard deviations from three different samples.

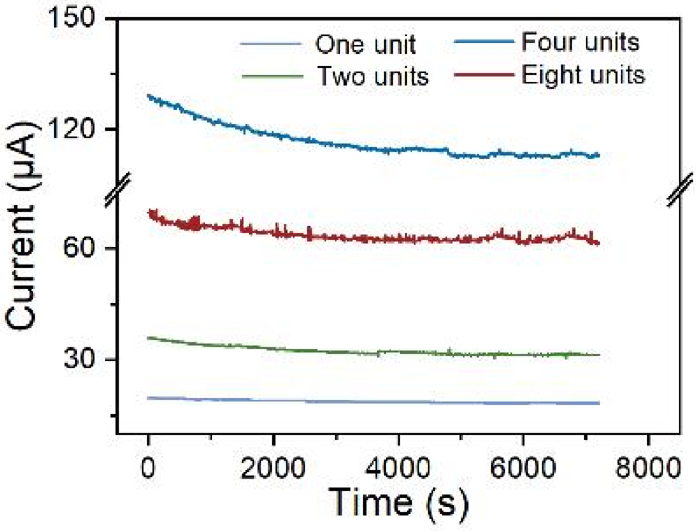

Supplementary Figure 32. Long-term open-circuit ionic currents in different numbers of Cu-TCPP membranes.

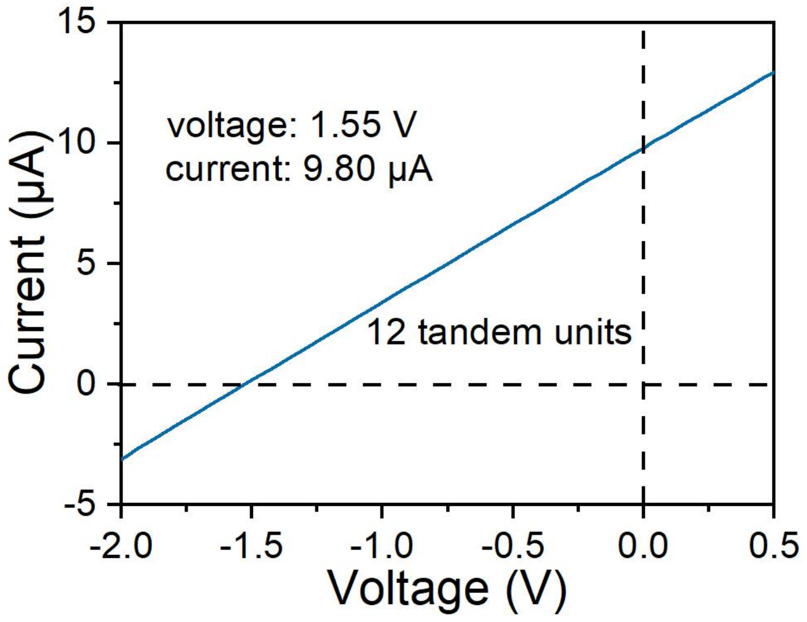

Supplementary Figure 33. I-V curve of the 12 tandem Cu-TCPP-RED stacks.

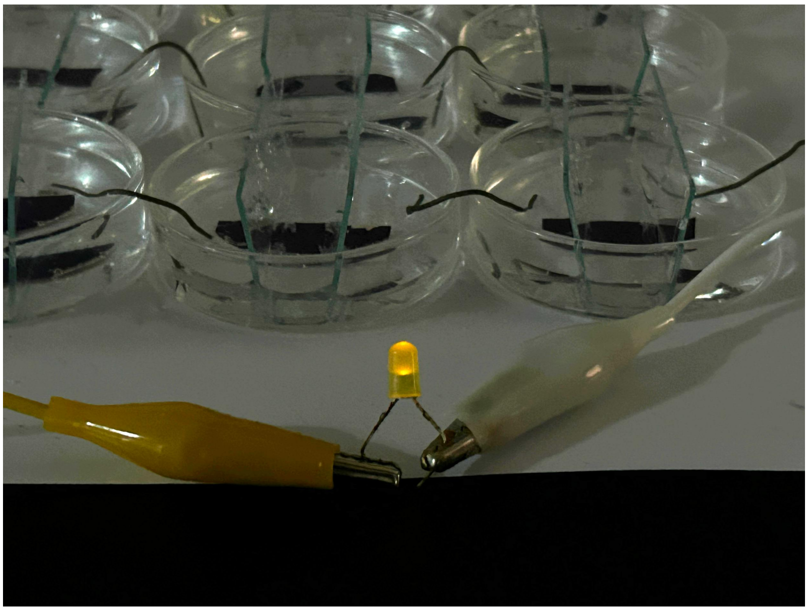

Supplementary Figure 34. Tandem Cu-TCPP-based-RED systems could directly power a LED lamp.

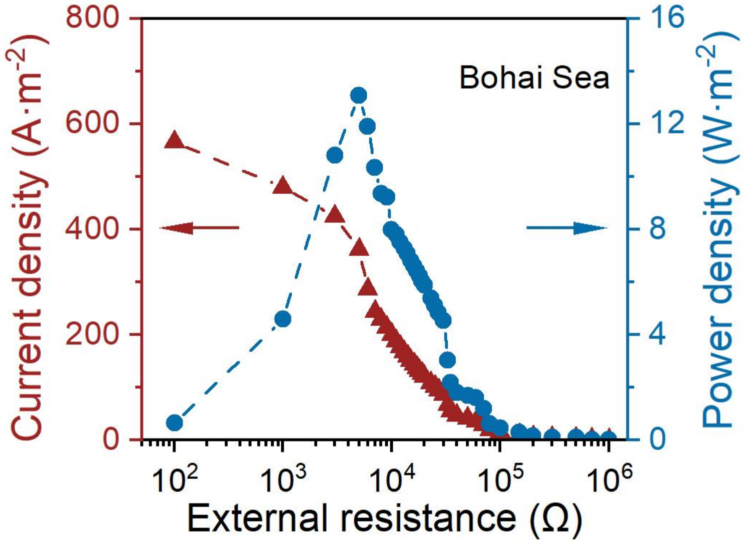

Supplementary Figure 35. Osmotic energy conversion performance of Cu-TCPP membrane using seawater from the Bohai Sea (China).

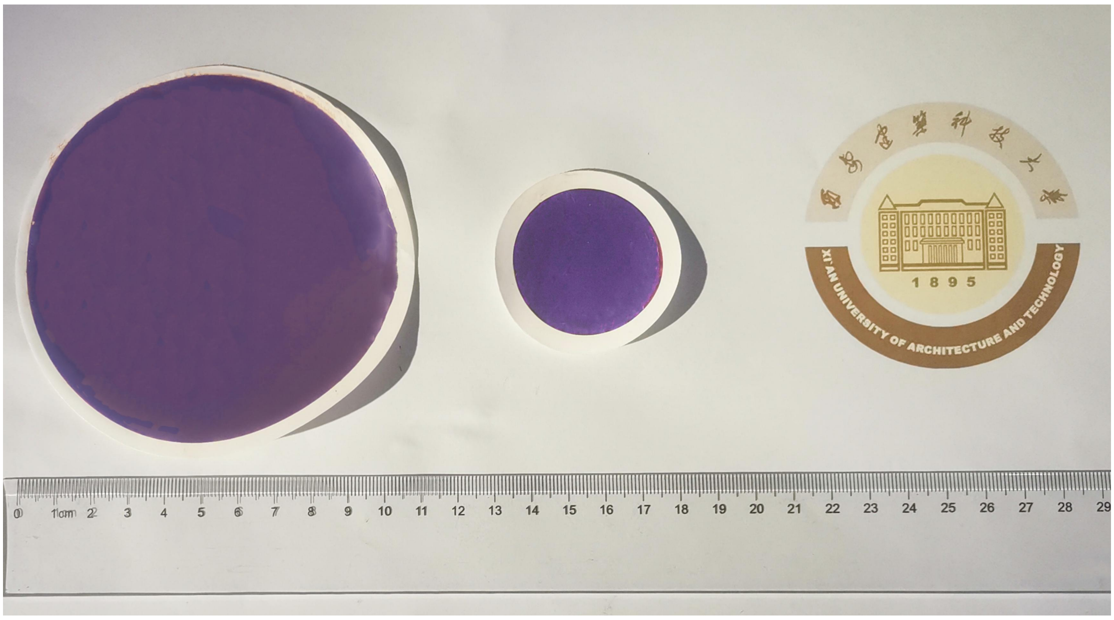

Supplementary Figure 36. Comparison images of different sizes of Cu-TCPP membrane.

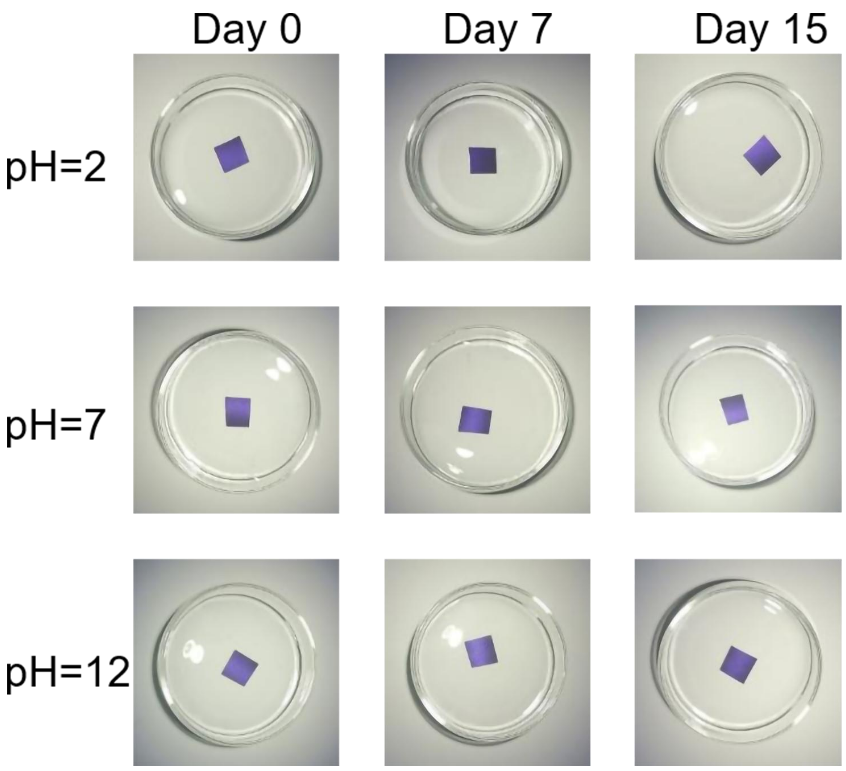

Supplementary Figure 37. Stability of Cu-TCPP membrane. Photos of the Cu-TCPP membrane samples soaked in an aqueous solution of pH 2, 7, and 12. The soaking time was 7 and 15 days.

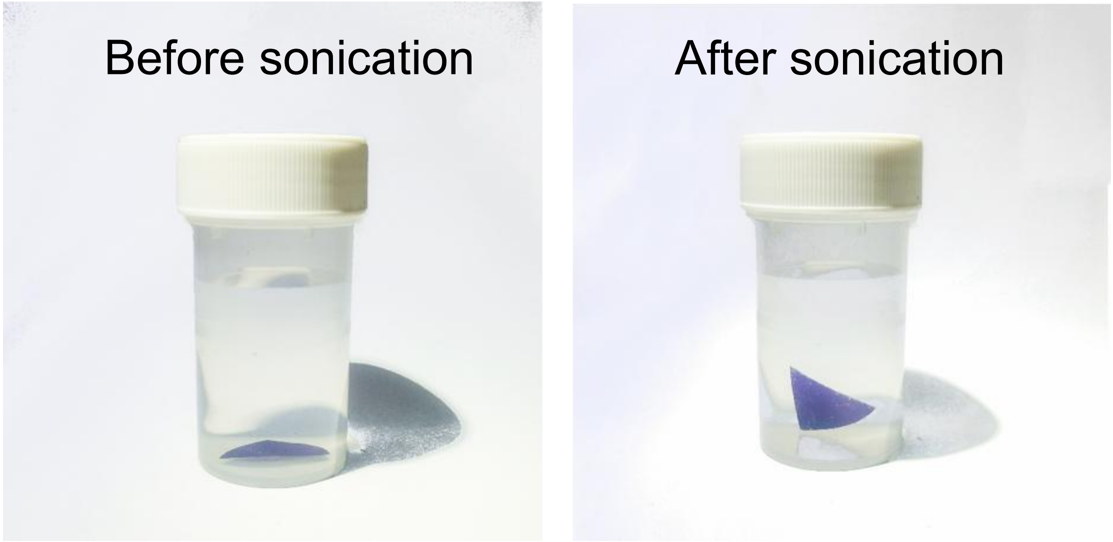

Supplementary Figure 38. Stability of Cu-TCPP membrane. The images showed the state of the Cu-TCPP membrane before and after 30 minutes sonication.

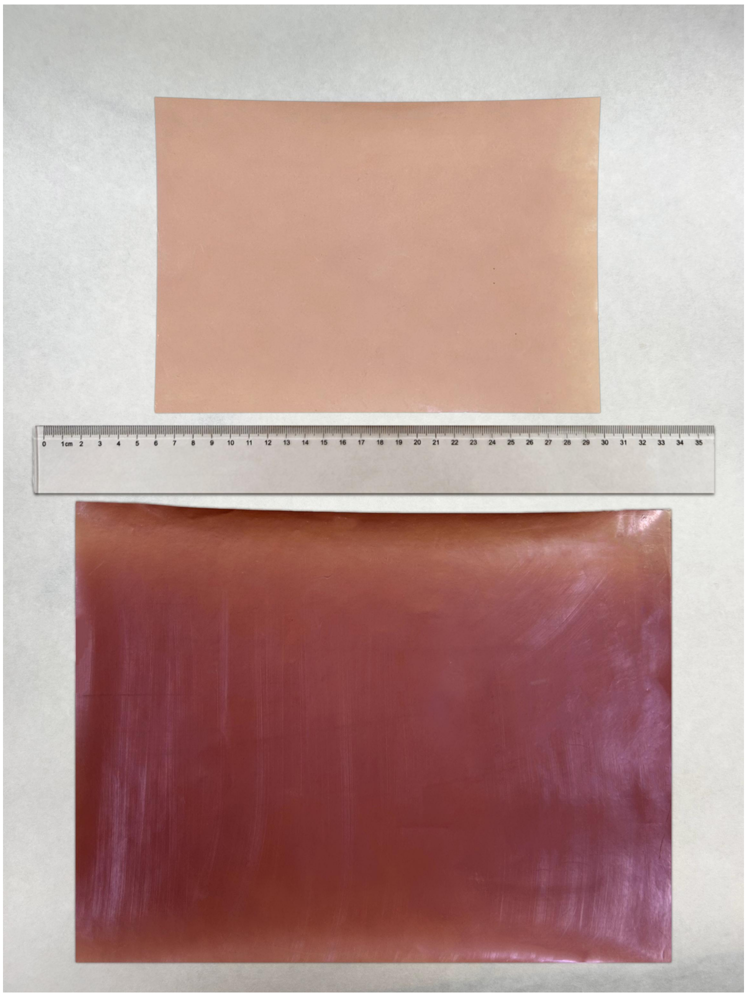

Supplementary Figure 39. Large-scale Cu-TCPP membrane (top: 300 cm^2, bottom: 600 cm^2) fabricated by spraying.

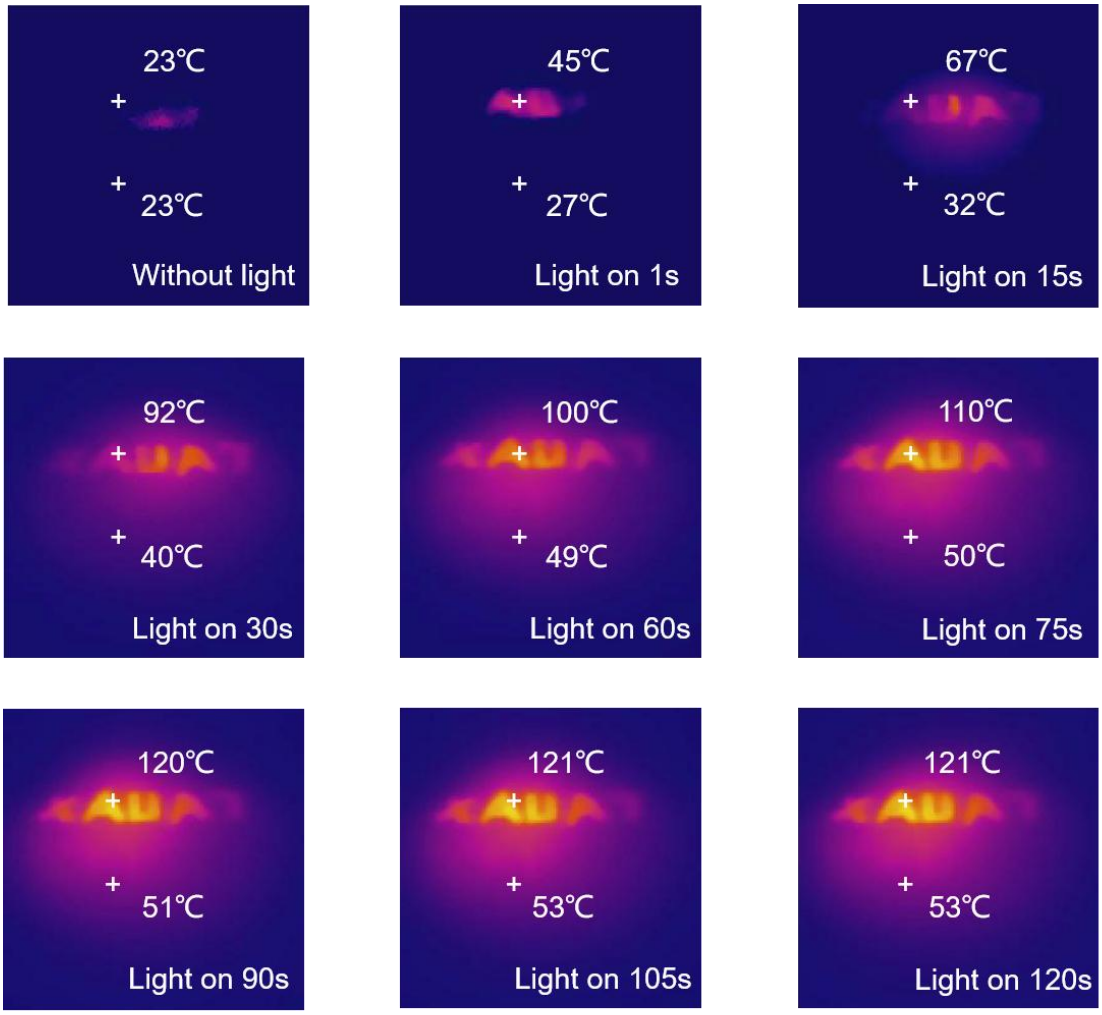

Supplementary Figure 40. Photo-thermal conversion phenomena. Infrared thermal images of Cu-TCPP and PVDF membrane under light irradiation taken at different time intervals.

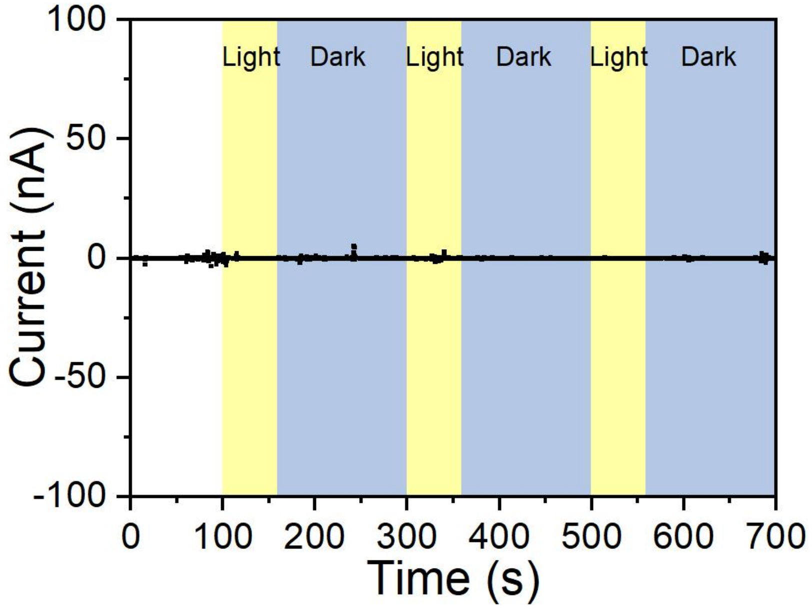

Supplementary Figure 41. Photo-response in PVDF membrane. No measurable photo-response was observed with PVDF membrane.

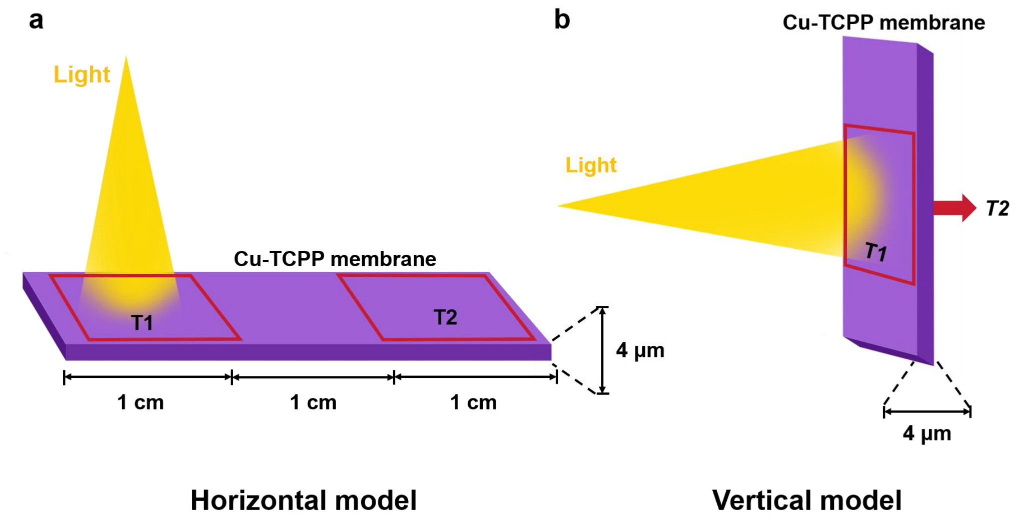

Supplementary Figure 42. Schematic of the temperature measurement for two models for under illumination. (a) In the horizontal model, the temperatures of illuminated and unilluminated ends were measured by infrared thermographic camera. (b) In the vertical model, the temperature of illuminated surface was measured by infrared thermographic camera, and the opposite side of the membrane was monitored by connecting to a thermalcouple.

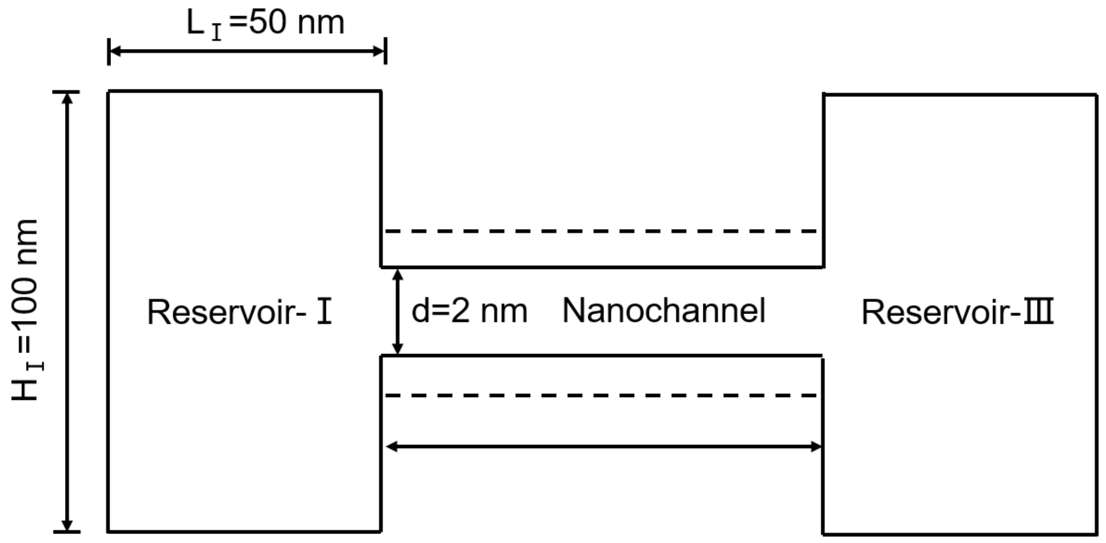

Supplementary Figure 43. Scheme of the simulation model channel. Two reservoirs (L_I =50 nm, H_I =100 nm) were connected by a charged channel (d = 2 nm and L = 100 nm). The surface charge density in the nanochannel was -60 mC/m^2. The concentration of the KCl solutions was set at 0.01 M.

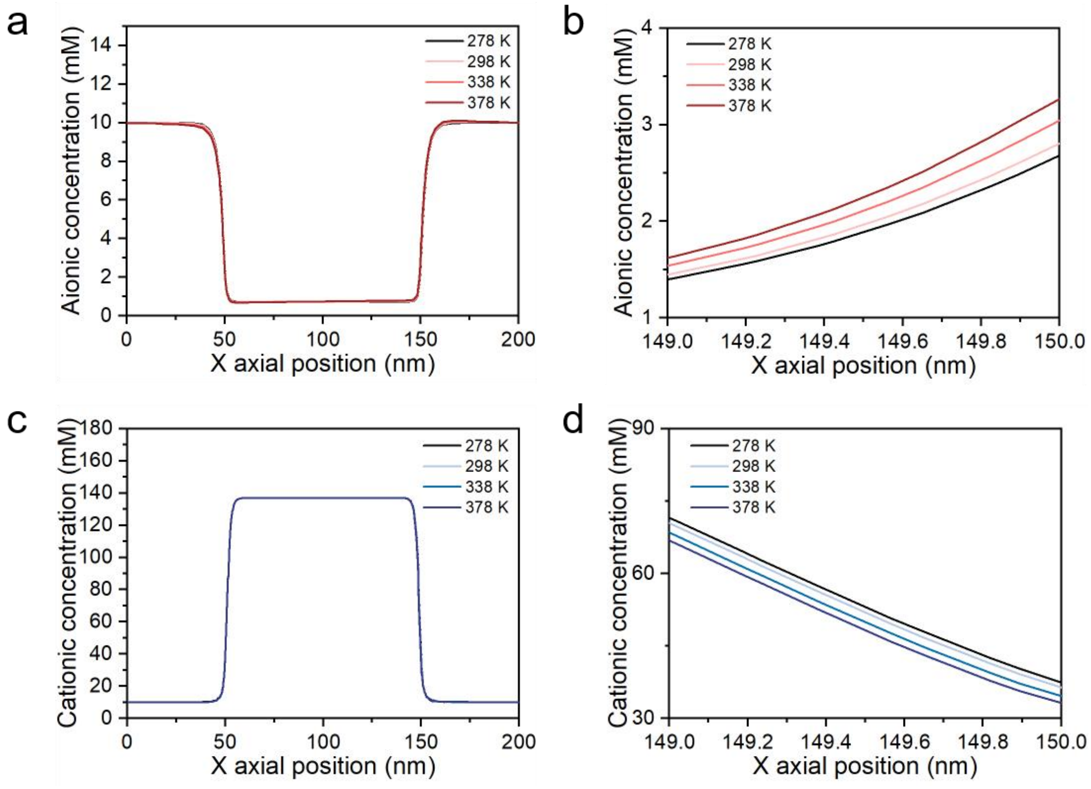

Supplementary Figure 44. Scheme of K^+ and Cl^- concentration profiles before and after the temperature change. Figures (b) and (d) depicted magnifications of the orifice of nanochannel/reservoir-III corresponding to figures (a) and (c), respectively. The temperature of reservoir-I and the nanochannel remained constant at 278 K, while reservoir-III increased from 278 K to 378 K. For simplicity, heat transfer was not taken into account in this study.

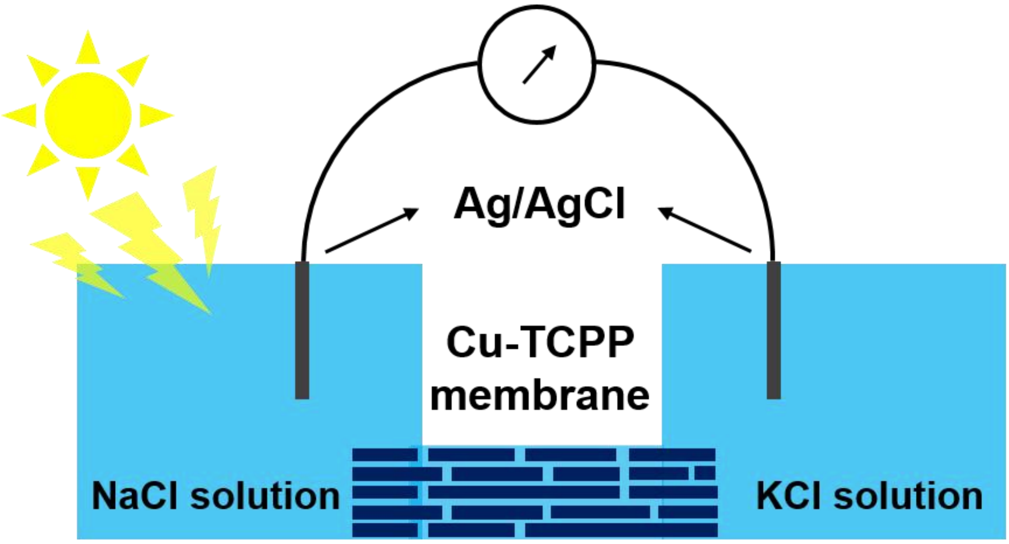

Supplementary Figure 45. Schematic diagram of K^+, Na^+ measurement by inductively coupled plasma (ICP) in the light.

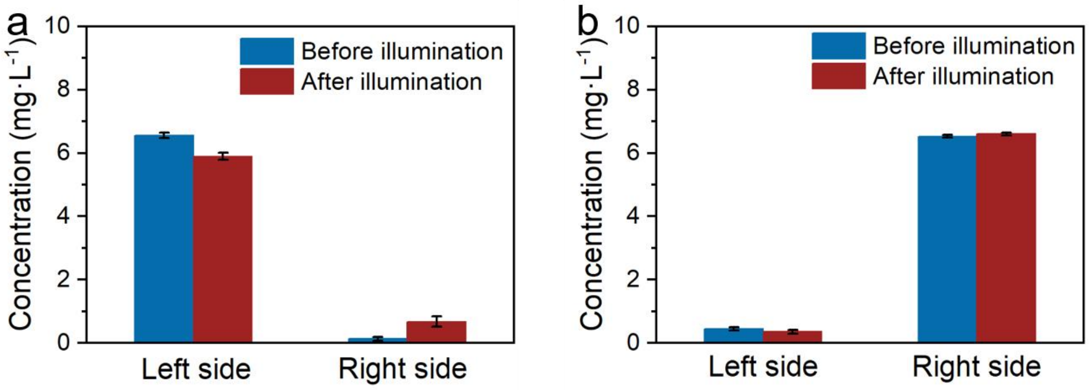

Supplementary Figure 46. The concentration of Na^+ (a) and K^+ (b) ions was measured by ICP before and after illumination. The error bars represent the standard deviations.

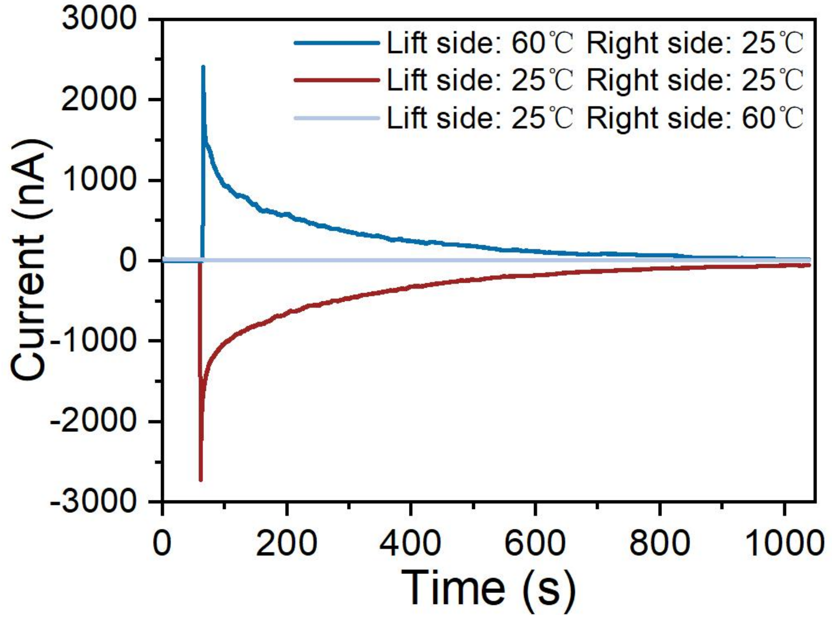

Supplementary Figure 47. Photothermal effect simulation. The electrolyte solution (KCl, 0.01M) added to both reservoirs is with equal concentration. Specifically, a heated KCl solution with a temperature value of approximately 60°C and a room-temperature solution was filled in the two reservoirs, respectively to simulate the photothermal experiment conditions. When the two reservoirs were without a temperature difference, the current value was almost zero. However, when the high-temperature solution was filled in Reservoir-I, a clear ionic current was observed in the same direction as that of the ionic current when Reservoir-I was illuminated.

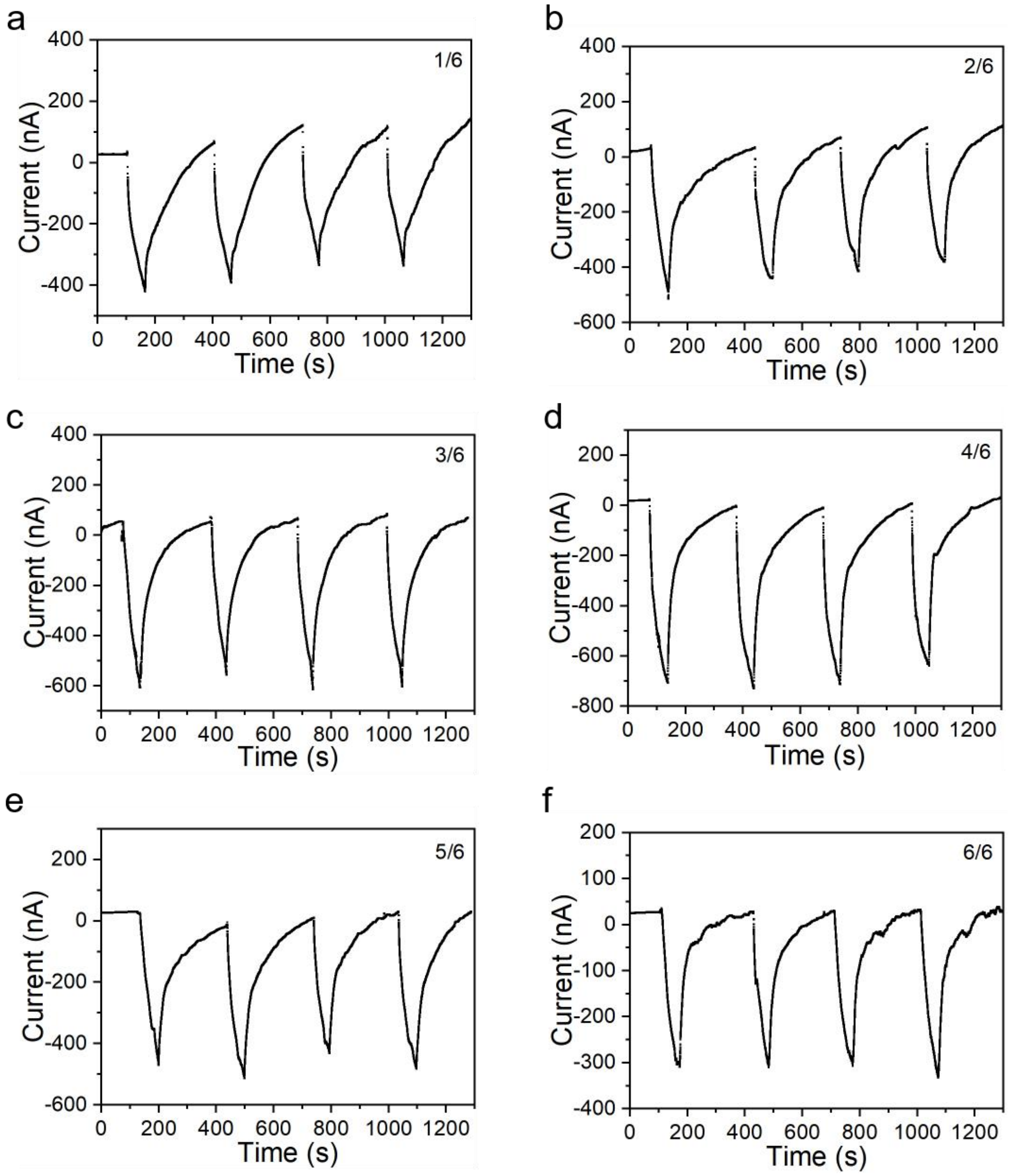

Supplementary Figure 48. Photo-responsive currents from different illuminated areas. (a~f) represent the ratio of the lighted area to the total area of 1/6~6/6.

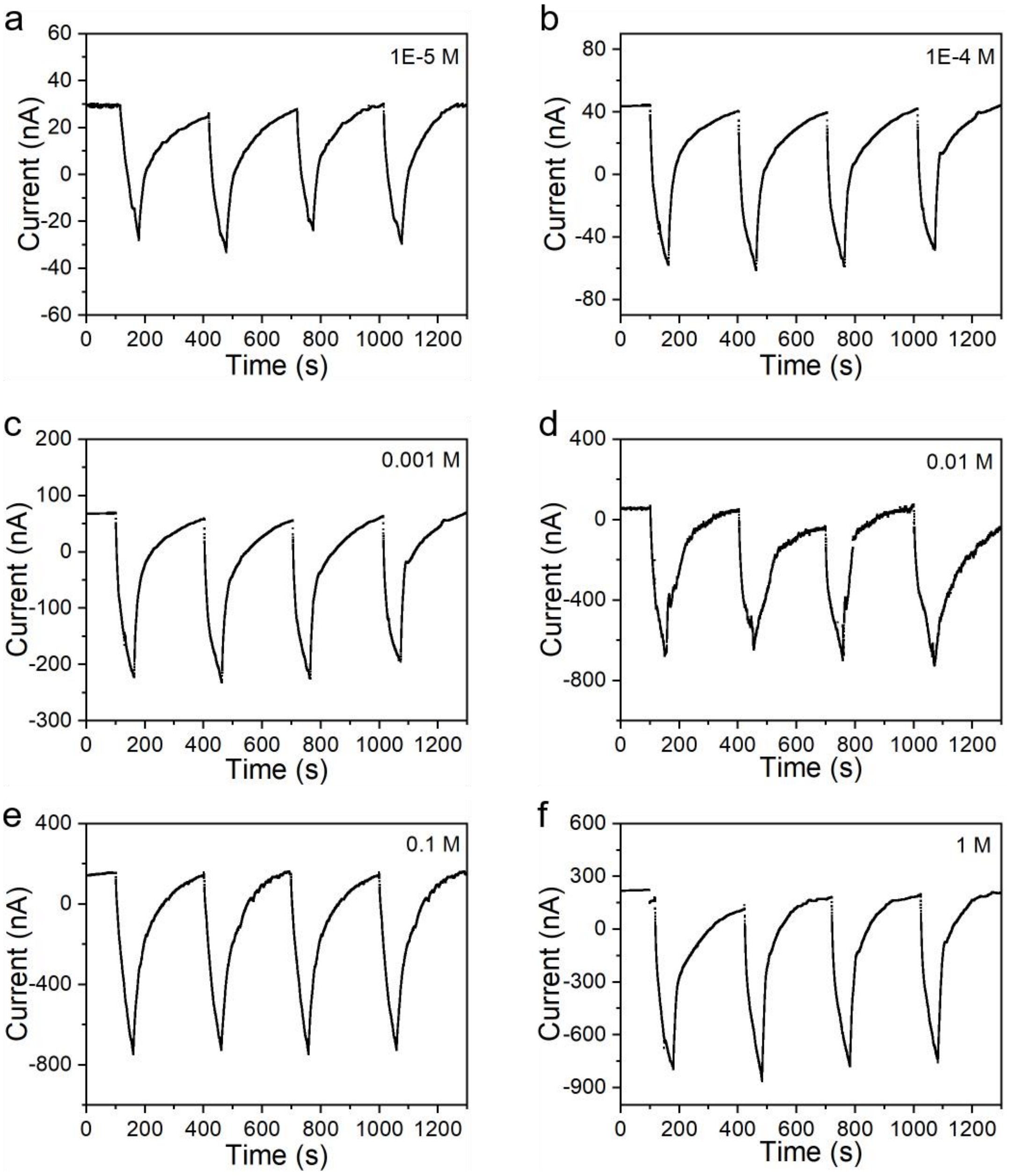

Supplementary Figure 49. The values of photocurrents generated at different concentrations. (a~f) represent the photo-responsive currents produced by 10^{-5}M~1M.

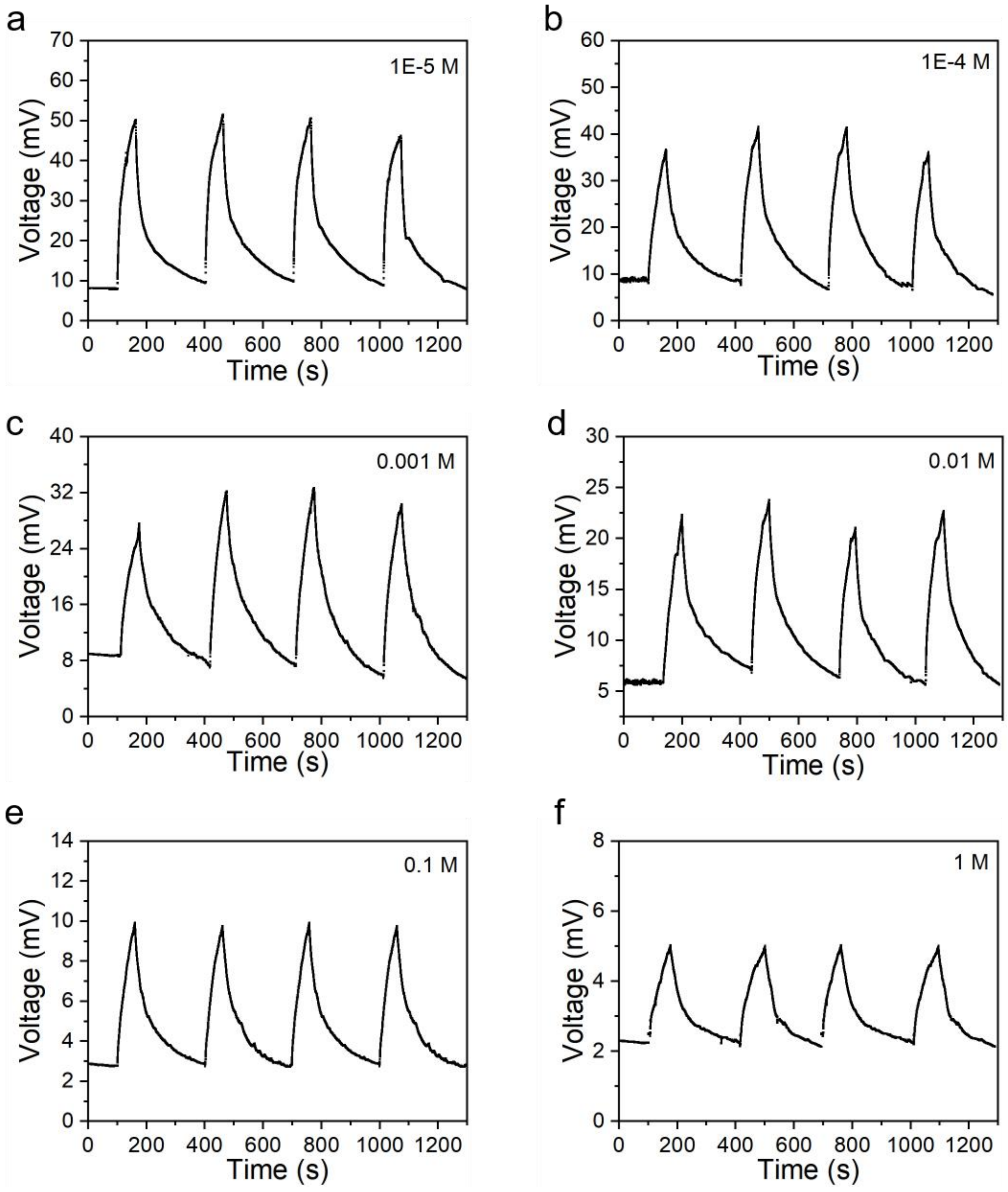

Supplementary Figure 50. The values of photovoltage generated at different concentrations. (a~f) represent the photo-responsive voltage produced by 10^{-5}M~1M.

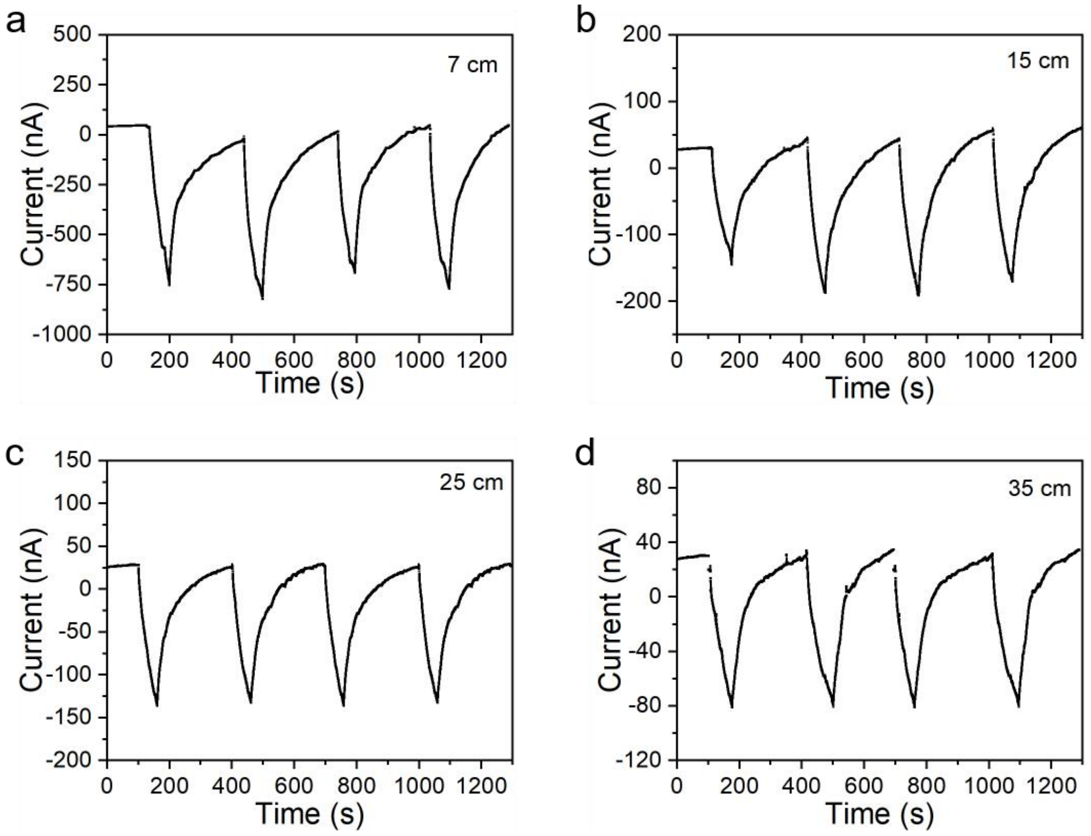

Supplementary Figure 51. The values of photocurrent resulting from different illumination distances. (a~d) represent the photo-responsive currents generated by the light source at 7~35 cm from the membrane surface.

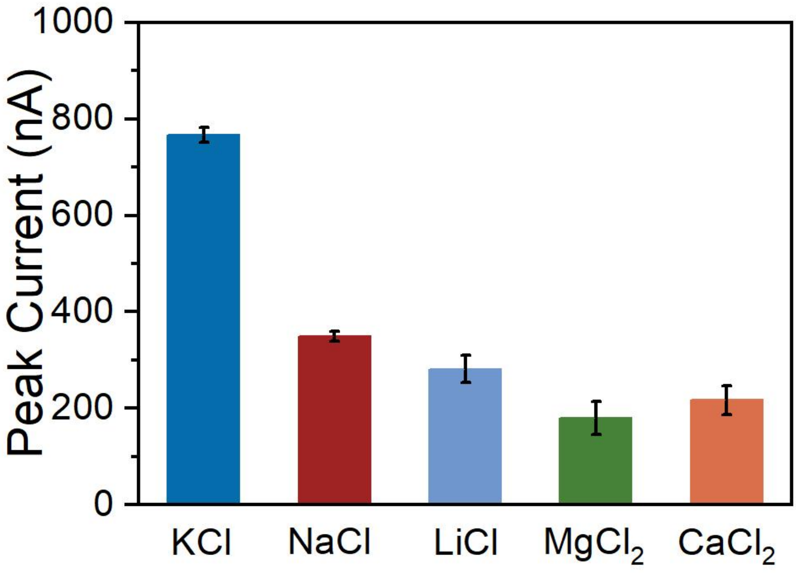

Supplementary Figure 52. Photo-responsive currents for different types of electrolyte solutions. Photocurrent testing was performed on electrolyte solutions (KCl, NaCl, LiCl, $MgCl_2$, $CaCl_2$) with the same concentration, which showed that the current generated by monovalent ions was generally higher than that of divalent ions due to the difference in hydration-free energy. The error bars represent the standard deviations.

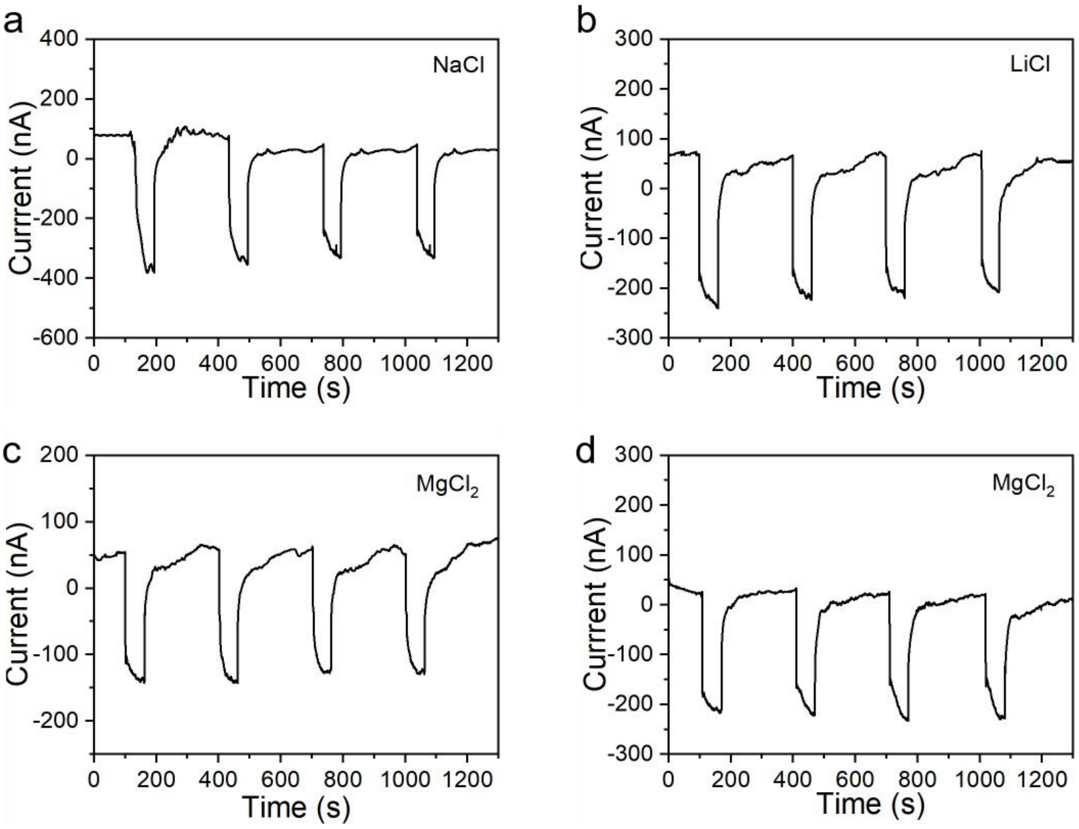

Supplementary Figure 53. The values of photocurrents generated by different kinds of electrolyte solutions. (a~d) represent the photo-responsive currents produced by different electrolyte solutions (NaCl, LiCl, $MgCl_2$, $CaCl_2$). The concentration of the tested electrolyte solutions was all 0.01 M.

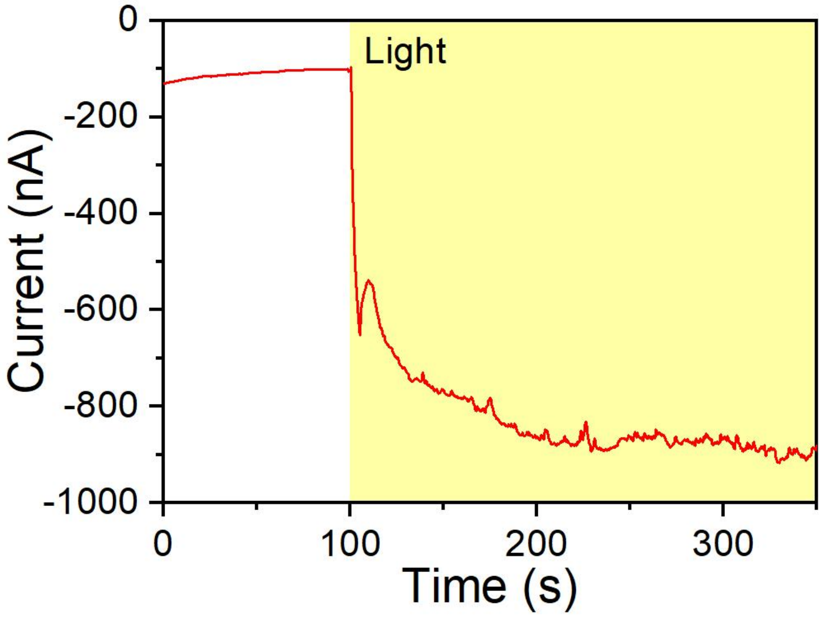

Supplementary Figure 54. Long-term light ionic current in Cu-TCPP membrane.

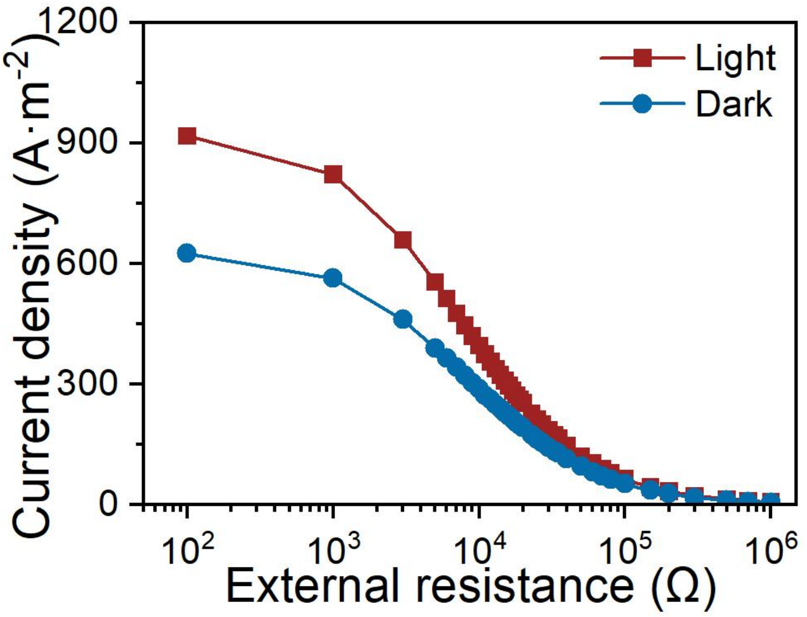

Supplementary Figure 55. Current density in artificial seawater/river water system before and after light irradiation.

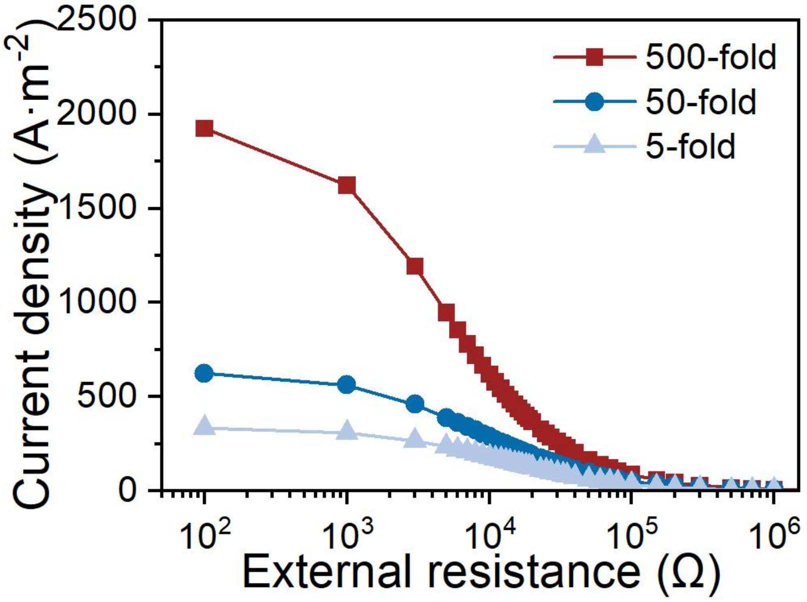

Supplementary Figure 56. The current density of Cu-TCPP membranes in different electrolyte solutions under light irradiation as a function of loading resistance. The NaCl solutions with different concentrations ranging from 0.05 ~ 5 M were used in Reservoir-I and 0.01 M in Reservoir-III.

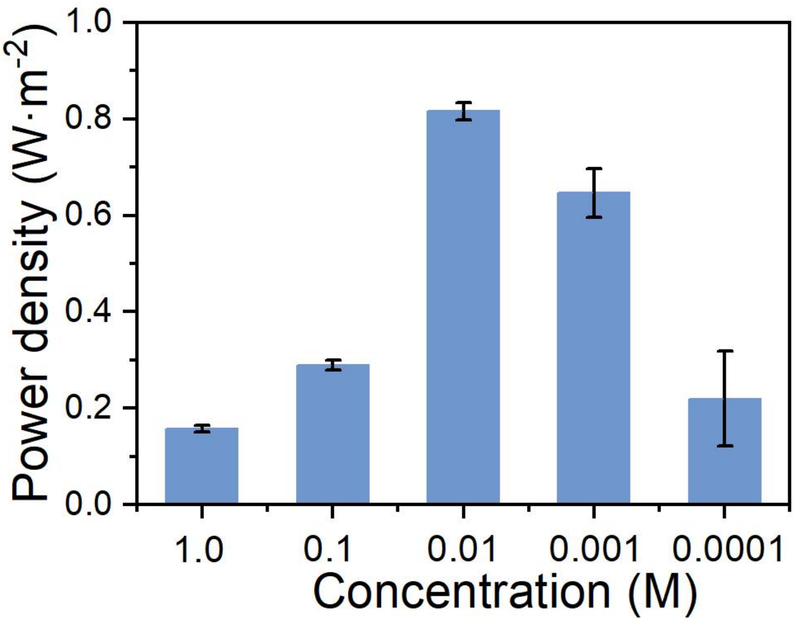

Supplementary Figure 57. Power density was produced by Cu-TCPP membranes at different concentrations under light irradiation. The error bars represent the standard deviations.

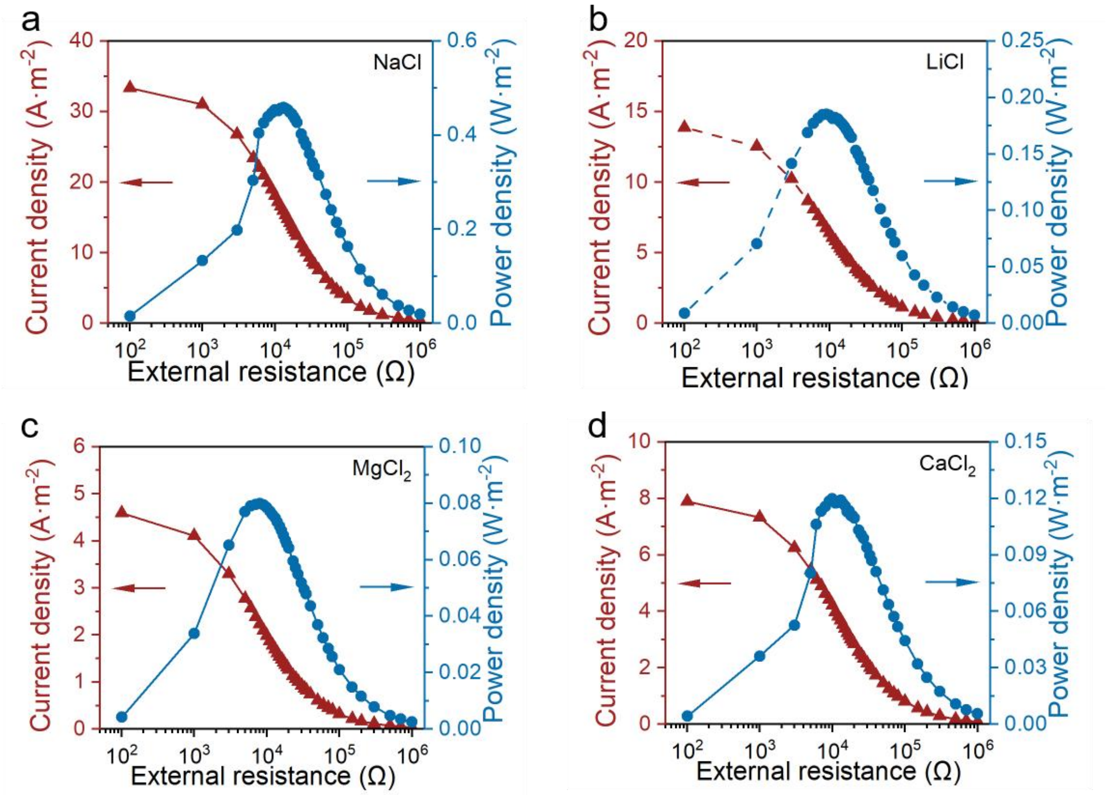

Supplementary Figure 58. The power density and current density generated by different types of electrolyte solutions (NaCl, LiCl, $MgCl_2$, $CaCl_2$) under light irradiation. The concentrations of the tested solutions were all 0.01 M.

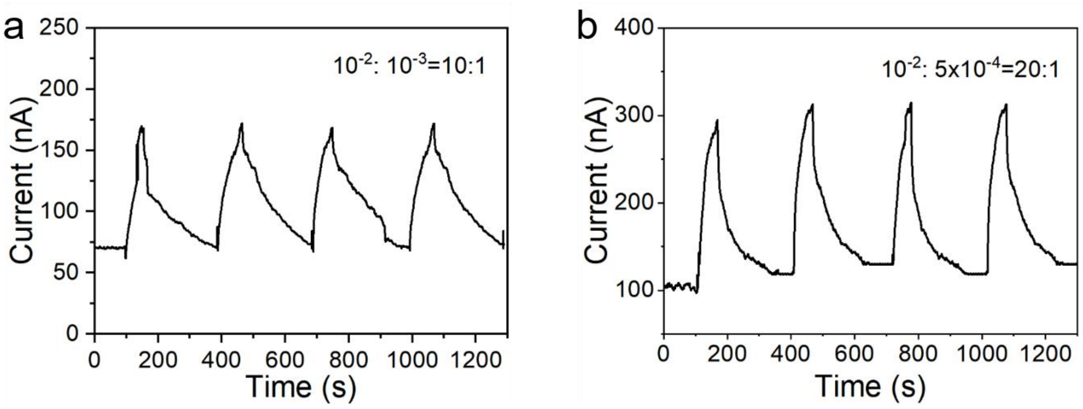

Supplementary Figure 59. The change in ion current with a concentration gradient was measured when light irradiated Reservoir-III. ($c_{Reservoir-I} > c_{Reservoir-III}$)

Supplementary Table 1. Comparison with reported osmotic energy conversion performance of nanofluidic membranes.

Concentration gradient	t_+	η (%)
2	0.82	21.2
5	0.88	29.7
20	0.90	32.1
50	0.87	28.6
100	0.88	29.1

Supplementary Table 2. Comparison with reported osmotic energy conversion performance of nanofluidic membranes.

Materials	The power density of artificial seawater/river water system (W m^{-2})	Resistance (kΩ)	Refs.
GO/Silk nanofiber membrane	5.07	35	1
WS_2@ANF composite membrane	6.01	23	2
MXene/PS-b-P2VP	6.74	5.5	3
MXene/Kevlar nanofiber composite	4.1	30	4
$Ti_3C_2T_x$ MXene membranes	4.6	5	5
bsGOM	5.5	36	6
PyPa-SO_3H/SANF membranes	8.3	5	7
$Ti_3C_2T_x$ MXene/BN (MXBN) membrane	2.3	60	8
2D metallic MoS_2	6.7	23	9
2D MOF/PAA	2.05	20	10
Graphene oxide membrane	0.77	10	11
BP/GO multilayer	3.4	31	12
Vertical GO	10.6	15	13
BN/ANF multilayer	5.9	10	14
Heterogeneous BCP/AAO	2.94	46	15
MOFs membrane	2.96	50	16
Polystyrene opals/AAO	3.16	20	17
Mesoporous carbon/AAO	3.46	10	18
Hydrogel membrane	3.9	23	19

Hydrogel-based nanofluidics	4.08	25	20
Ultrasmall silica nanochannels	1	20	21
COF-LZU1@CNT-CNF	4.26	45	22
Silica/AAO heterostructure membranes	4.5	10	23
WO_3/AAO-ZIF-8	1.93	40	24
SPEEK/AAO	4.8	30	25
Mesoporous carbon-silica/AAO	5.04	10	26
Cyto-compatible asymmetric polypyrrole	0.087	5	27
TFP-TPA COF@ANM	5.41	15	28
Carbonaceous nanowires /AAO	2.78	13	29
SPES/ImPES	5.8	10	30
COF-TpPa-SO_3H	5.9	23	31
TpPa-SO3H COF	5.9	30	32
Bacterial cellulose membranes	0.23	7	33
SPAEK/PES	4.8	30	34
PS-b-P4VP/PEO-b-PMA	2.66	10	35

Polymer/MOF	2.87	6.5	36
Asymmetric hydrogel membrane	7.87	10	37
Porphyrin/Al_2O_3	2.16	10	38
Nanochannel array	13.2	10	39
Cu-TCPP membrane without light irradiation	15.54	9	This work
Cu-TCPP membrane with light irradiation	31.93	8	This work

Supplementary Table 3. List of parameters for numerical simulation model.

Parameter	Description	Value
A	Length of reservoir	100 nm
B	Width of reservoir	100 nm
D	Diameter of nanochannel	10 nm
L	Length of nanochannel	25 nm
σ	Surface charge density	-0.06 C m^{-2}
$d1$	Interlayer spacing	2 nm
$d2$	Width of nanosheet	0.5 nm
D_p	Diffusion coefficient of K^+	1.957×10^{-9} m^2 s^{-1}
D_n	Diffusion coefficient of Cl^-	2.032×10^{-9} m^2 s^{-1}
$c0$	Electrolyte concentration	1-50 mM
T	Temperature	298-378 K

Supplementary Table 4. Thickness and width of membrane for different test areas.

Testing area (10^{-2} mm^2)	Thickness (μm)	Width (cm)
1	2	0.5
2	4	0.5
4	8	0.5
10	20	0.5
20	20	1.0
40	20	2.0
60	20	3.0

Supplementary Table 5. Cation content of seawater.

Type	Na (mg L^{-1})	K (mg L^{-1})	Mg (mg L^{-1})	Ca (mg L^{-1})
Bohai Sea	9350	334	1056	371

Supplementary Table 6. Price List of materials elected commercial membranes.

Material	Company	Price
$Cu(NO_3)_2\cdot3H_2O$	Macklin, China	0.11 CNY g^{-1}

Tetra-(4-carboxyphenyl) porphyrin	Macklin, China	76 CNY g^{-1}
N,N-Dimethylformamide	Aladdin, China	45.36 CNY L^{-1}
Ethanol	Greagent, China	14.31 CNY L^{-1}
**Membrane**	**Company**	**Price**
Fumasep FKD	Fumatech, Germany	16633 CNY m^{-2}
Selemion CMV	Asahi Glass Company, Japan	30000 CNY m^{-2}
Fuji CEM-II	Fujifilm, Japan	1487.6 CNY m^{-2}
Nafion 117	Dupont, Amercica	22950.8 CNY m^{-2}
Cu-TCPP membrane	This work	312.20 CNY m^{-2}

The data of $Cu(NO_3)_2 \cdot 3H_2O$ and tetra-(4-carboxyphenyl) porphyrin were collected from www.macklin.cn, visited in Oct. 23th, 2023. The data of DMF were collected from www.aladdin-e.com, visited in Oct. 23th, 2023. The data of ethanol were collected from titansci.com, visited in Oct. 23th, 2023. The prices of commercial cation exchange membranes were collected from www.1688.com, visited in Oct. 23th, 2023.

References

1. Xin W*, et al.* Biomimetic nacre-like silk-crosslinked membranes for osmotic energy harvesting. *ACS Nano* **14**, 9701-9710 (2020).

2. Wang Q*, et al.* Efficient Solar-osmotic Power Generation from Bioinspired Anti-fouling 2D WS_2 Composite Membranes. *Angew. Chem. Int. Ed.*, e202302938 (2023).

3. Lin X*, et al.* Heterogeneous MXene/PS-b-P2VP nanofluidic membranes with controllable ion transport for osmotic energy conversion. *Adv. Funct. Mater.* **31**, 2105013 (2021).

4. Zhang Z, Yang S, Zhang P, Zhang J, Chen G, Feng X. Mechanically strong MXene/Kevlar nanofiber composite membranes as high-performance nanofluidic osmotic power generators. *Nat. Commun.* **10**, 2920 (2019).

5. Ding L*, et al.* Oppositely charged $Ti_3C_2T_x$ MXene membranes with 2D nanofluidic channels for osmotic energy harvesting. *Angew. Chem. Int. Ed.* **132**, 8798-8804 (2020).

6. Qian Y*, et al.* Boosting osmotic energy conversion of graphene oxide membranes via self-exfoliation behavior in nano-confinement spaces. *J. Am. Chem. Soc.* **144**, 13764-13772 (2022).

7. Man Z*, et al.* Serosa-mimetic nanoarchitecture membranes for highly efficient osmotic energy generation. *J. Am. Chem. Soc.* **143**, 16206-16216 (2021).

8. Yang G*, et al.* Stable $Ti_3C_2T_x$ MXene–Boron Nitride Membranes with Low Internal Resistance for Enhanced Salinity Gradient Energy Harvesting. *ACS Nano* **15**, 6594-6603 (2021).

9. Zhu C*, et al.* Metallic two-dimensional MoS2 composites as high-performance osmotic energy conversion membranes. *J. Am. Chem. Soc.* **143**, 1932-1940 (2021).

10. Wang C, Liu FF, Tan Z, Chen YM, Hu WC, Xia XH. Fabrication of Bio‐Inspired 2D MOFs/PAA Hybrid Membrane for Asymmetric Ion Transport. *Adv. Funct. Mater.* **30**, 1908804 (2020).

11. Ji J*, et al.* Osmotic power generation with positively and negatively charged 2D nanofluidic membrane pairs. *Adv. Funct. Mater.s* **27**, 1603623 (2017).

12. Zhang Z*, et al.* Oxidation promoted osmotic energy conversion in black phosphorus

membranes. *Proceedings of the Nat. Academy of Sciences* **117**, 13959-13966 (2020).

13. Zhang Z*, et al.* Vertically transported graphene oxide for high‐performance osmotic energy conversion. *Adv. Sci.* **7**, 2000286 (2020).

14. Chen C*, et al.* Bio-inspired nanocomposite membranes for osmotic energy harvesting. *Joule* **4**, 247-261 (2020).

15. Sui X*, et al.* Engineered nanochannel membranes with diode-like behavior for energy conversion over a wide pH range. *ACS Appl. Mater. Interfaces* **11**, 23815-23821 (2018).

16. Liu Y-C, Yeh L-H, Zheng M-J, Wu KC-W. Highly selective and high-performance osmotic power generators in subnanochannel membranes enabled by metal-organic frameworks. *Sci. Adv.* **7**, eabe9924 (2021).

17. Xiao T*, et al.* Tunable rectifications in nanofluidic diodes by ion selectivity of charged polystyrene opals for osmotic energy conversion. *J. Mater. Chem. A* **8**, 11275-11281 (2020).

18. Gao J, Guo W, Feng D, Wang H, Zhao D, Jiang L. High-performance ionic diode membrane for salinity gradient power generation. *J. Am. Chem. Soc.* **136**, 12265-12272 (2014).

19. Zhang Z, He L, Zhu C, Qian Y, Wen L, Jiang L. Improved osmotic energy conversion in heterogeneous membrane boosted by three-dimensional hydrogel interface. *Nat. Commun.* **11**, 875 (2020).

20. Chen W*, et al.* Improved ion transport in hydrogel-based nanofluidics for osmotic energy conversion. *ACS Cent. Sci.* **6**, 2097-2104 (2020).

21. Chen K, Yao L, Yan F, Liu S, Yang R, Su B. Thermo-osmotic energy conversion and storage by nanochannels. *J. Mater. Chem. A* **7**, 25258-25261 (2019).

22. Li R, Zhai J, Jiang J, Wang Q, Wang S. Improved Interfacial Ion Transport through Nanofluidic Hybrid Membranes Based on Covalent Organic Frameworks for Osmotic Energy Generation. *ACS Appl. Energy Mater.* **5**, 7176-7184 (2022).

23. Xin W*, et al.* High-performance silk-based hybrid membranes employed for osmotic energy conversion. *Nat. Commun.* **10**, 3876 (2019).

24. Fu L, Wang Y, Jiang J, Lu B, Zhai J. Sandwich "Ion Pool"-Structured Power Gating for Salinity Gradient Generation Devices. *ACS Appl. Mater. Interfaces* **13**, 35197-35206

(2021).

25. Hou S*, et al.* Charged porous asymmetric membrane for enhancing salinity gradient energy conversion. *Nano Energy* **79**, 105509 (2021).

26. Zhou S*, et al.* Interfacial super‐assembly of ordered mesoporous carbon‐silica/AAO hybrid membrane with enhanced permselectivity for temperature‐and pH‐sensitive smart ion transport. *Angew. Chem. Int. Ed.* **133**, 26371-26380 (2021).

27. Yu C*, et al.* A smart cyto-compatible asymmetric polypyrrole membrane for salinity power generation. *Nano Energy* **53**, 475-482 (2018).

28. Gao M*, et al.* A bioinspired ionic diode membrane based on sub-2 nm covalent organic framework channels for ultrahigh osmotic energy generation. *Nano Energy* **105**, 108007 (2023).

29. Xie L*, et al.* Sequential superassembly of nanofiber arrays to carbonaceous ordered mesoporous nanowires and their heterostructure membranes for osmotic energy conversion. *J. Am. Chem. Soc.* **143**, 6922-6932 (2021).

30. Sun Y*, et al.* Tailoring a poly (ether sulfone) bipolar membrane: osmotic‐energy generator with high power density. *Angew. Chem. Int. Ed.* **59**, 17423-17428 (2020).

31. Hou S, Ji W, Chen J, Teng Y, Wen L, Jiang L. Free‐Standing Covalent Organic Framework Membrane for High‐Efficiency Salinity Gradient Energy Conversion. *Angew. Chem. Int. Ed.* **133**, 10013-10018 (2021).

32. Zuo X*, et al.* Thermo‐Osmotic Energy Conversion Enabled by Covalent‐Organic‐Framework Membranes with Record Output Power Density. *Angew. Chem. Int. Ed.* **61**, e202116910 (2022).

33. Wu Z*, et al.* Oppositely charged aligned bacterial cellulose biofilm with nanofluidic channels for osmotic energy harvesting. *Nano Energy* **80**, 105554 (2021).

34. Zhu X*, et al.* Unique ion rectification in hypersaline environment: A high-performance and sustainable power generator system. *Sci. Adv.* **4**, eaau1665 (2018).

35. Zhang Z*, et al.* Ultrathin and ion-selective Janus membranes for high-performance osmotic energy conversion. *J. Am. Chem. Soc.* **139**, 8905-8914 (2017).

36. Li R, Jiang J, Liu Q, Xie Z, Zhai J. Hybrid nanochannel membrane based on polymer/MOF for high-performance salinity gradient power generation. *Nano Energy*

53, 643-649 (2018).

37. Bian G*, et al.* Anti - swelling gradient polyelectrolyte hydrogel membranes as high - performance osmotic energy generators. *Angew. Chem. Int. Ed.* **133**, 20456-20462 (2021).
38. Zhang D, Ren Y, Fan X, Zhai J, Jiang L. Photoassisted salt-concentration-biased electricity generation using cation-selective porphyrin-based nanochannels membrane. *Nano Energy* **76**, 105086 (2020).
39. Li C, Wen L, Sui X, Cheng Y, Gao L, Jiang L. Large-scale, robust mushroom-shaped nanochannel array membrane for ultrahigh osmotic energy conversion. *Sci. Adv.* **7**, eabg2183 (2021).
