# Supplementary material for: Light-responsive and ultrapermeable two-dimensional metal-organic framework membrane for efficient ionic energy harvesting
Source: Nat Commun. 2024 Mar 8;15:2125. doi: 10.1038/s41467-024-46439-w (PMC10923900; doi:10.1038/s41467-024-46439-w)
Supplement: Supplementary file 3 — Description of Additional Supplementary Files [file 41467_2024_46439_MOESM3_ESM.pdf]

Description of Additional Supplementary Files

File Name: Supplementary Movie 1
Description: Twelve Cu-TCPP-RED unit cells connected in series produce a stable voltage for calculator to work.

File Name: Supplementary Movie 2
Description: Anti-gradient ion transport behavior driven by natural sunlight.
